# Transcriptome Analysis Reveals the Contribution of Thermal and the Specific Effects in Cellular Response to Millimeter Wave Exposure

**DOI:** 10.1371/journal.pone.0109435

**Published:** 2014-10-10

**Authors:** Denis Habauzit, Catherine Le Quément, Maxim Zhadobov, Catherine Martin, Marc Aubry, Ronan Sauleau, Yves Le Dréan

**Affiliations:** 1 Transcription, Environment and Cancer group, IRSET - Institute of Research in Environmental and Occupational Health, INSERM U1085, University of Rennes 1, Rennes, France; 2 Institute of Electronics and Telecommunications of Rennes - IETR, University of Rennes 1, UMR CNRS 6164, Rennes, France; 3 Plate-forme Génomique Santé, Biosit, Université de Rennes 1, Rennes, France; National Research Council, Italy

## Abstract

Radiofrequency radiations constitute a new form of environmental pollution. Among them, millimeter waves (MMW) will be widely used in the near future for high speed communication systems. This study aimed therefore to evaluate the biocompatibility of MMW at 60 GHz. For this purpose, we used a whole gene expression approach to assess the effect of acute 60 GHz exposure on primary cultures of human keratinocytes. Controls were performed to dissociate the electromagnetic from the thermal effect of MMW. Microarray data were validated by RT-PCR, in order to ensure the reproducibility of the results. MMW exposure at 20 mW/cm^2^, corresponding to the maximum incident power density authorized for public use (local exposure averaged over 1 cm^2^), led to an increase of temperature and to a strong modification of keratinocyte gene expression (665 genes differentially expressed). Nevertheless, when temperature is artificially maintained constant, no modification in gene expression was observed after MMW exposure. However, a heat shock control did not mimic exactly the MMW effect, suggesting a slight but specific electromagnetic effect under hyperthermia conditions (34 genes differentially expressed). By RT-PCR, we analyzed the time course of the transcriptomic response and 7 genes have been validated as differentially expressed: *ADAMTS6, NOG, IL7R, FADD, JUNB, SNAI2* and *HIST1H1A*. Our data evidenced a specific electromagnetic effect of MMW, which is associated to the cellular response to hyperthermia. This study raises the question of co-exposures associating radiofrequencies and other environmental sources of cellular stress.

## Introduction

Radiofrequencies (RF) constitute a new source of anthropogenic pollution and there is an increasing concern about their potential health effects on human beings. RF are electromagnetic waves ranging from 30 kHz to 300 GHz, which are used in many applications (wireless communications, detection and localization, medicine, power transfer, *etc*.). In 2011, these radiations were classified by the International Agency for Research on Cancer (IARC) as “possibly carcinogenic to humans” (group 2B) [1], [2]. This classification is a consequence of epidemiologic studies, especially from the international INTERPHONE consortium and from the Hardell's group [3], [4], [5], [6]. However, this classification is currently controversial [7], because no *in vivo* or *in vitro* studies had confirmed this fact, or proposed possible mechanisms. Additional studies are therefore needed to clarify the situation.

Environmental RF exposures evolve with wireless technologies and the current saturation of the lower part of the electromagnetic spectrum induces a demand for new frequency ranges. The millimeter waves (MMW), corresponding to the frequencies between 30 GHz and 300 GHz, have been identified as highly promising for the next generation of broadband wireless communications. MMW, especially the 60-GHz band, possess several advantages and some applications are already on the market. First, they allow faster data rates. Second, they lead secured wireless communications due to the high atmospheric absorption. MMW are also used in other applications, such as Active Denial Systems (non-lethal weapon) [8], body scanners mainly used in the airports [9], and radar systems (collision avoidance, radio astronomy, police and military radars) [10]. Interestingly, MMW have been used for medical therapy in eastern European countries [11], [12], indicating that these radiations have an effect on human body. Therefore, they may constitute a risk, and their effects need to be carefully evaluated before their widespread use by the general public.

Three frequencies are used in therapy: 42.2, 53.6 and 61.2 GHz. These radiations, generally in association with other treatments, gave positive clinical results in the cure of miscellaneous diseases, such as ulcers, pain relief, cardiovascular diseases, wound healing, bronchial asthma, skin disorders or cancers [12]. Meanwhile, it was demonstrated that MMW may have medical effect on inflammatory [13], [14], [15] and analgesic [16], [17], [18] responses. The mechanism involved in MMW biological effects remains to be elucidated, especially because these radiations have a shallow penetration (<1 mm) [19]. This suggests that the main target of MMW is the skin. MMW bioeffects must be initiated through secreted factors by the skin cells and/or through the nerve endings of the peripheral nervous system.

As a consequence of the MMW shallow penetration, the electromagnetic energy is absorbed by low quantity of biological material, leading to relatively high levels of the specific absorption rates (SAR), compared to the lower part of the RF spectrum. As MMW belong to the microwave family, this energy transfer induces a heat effect for the incident power densities (IPD) above 5 mW/cm^2^ [18], [19], [20]. This thermal effect is currently the main well-established biological effect and served as a basis for the definition of the MMW exposure standards and guidelines by the International Commission on Non-Ionizing Radiation Protection (ICNIRP). The recommended ICNIRP limits depend on the MMW exposure scenarios. First, when user is far from the MMW source, the IPD is limited to 1 mW/cm^2^ for the general public (IPD averaged over 20 cm^2^ of exposed tissue). Second, when the MMW source is closed to the user or directly on the user's skin and generates a very restricted exposure area, the IPD (averaged over 1 cm^2^ of exposed tissue) is then limited to 20 mW/cm^2^ [21]. These are the maximum permissible exposure levels applied for instance to the local exposure in the body-centric wireless networks [22].

This study aimed to evaluate biological effects in the 60-GHz band, under local exposure conditions, where the IPD could reach 20 mW/cm^2^. In such exposure scenarios, MMWs will generate a local increase of temperature [20]. Therefore, the global cellular response will integrate two effects: the first one linked to the heat and a putative second one linked to the electromagnetic field. Our previous *in vitro* studies, have shown that MMW at 57–64 GHz have no effect on protein homeostasis, for IPD low enough (0.14 mW/cm^2^) to avoid any increase of temperature (ΔT<0.05°C) [23], [24]. Furthermore, under athermal condition, whole transcriptome analyses showed a very limited transient modification of gene expression for an IPD close to 1 mW/cm^2^ [25]. To address the biocompatibility issues, under representative body-centric wireless networks exposure scenarios, we performed a large-scale genomic analysis on primary human keratinocyte cells exposed to MMW at 20 mW/cm^2^, that is equivalent to general public IPD limits for this future application.

## Materials and Methods

### Cell culture

Primary human keratinocytes were obtained from Invitrogen (Saint-Aubin, France), Cells were pooled and isolated from 3 neonatal foreskin circumcised patients, and they were maintained as advised by supplier. Briefly, cells were cultured onto collagen IV coated plates (Becton Dikinson Franklin Lakes, NJ), in supplemented Keratinocyte-SFM medium (Gibco, Carlsbad, CA) with antibiotics (Invitrogen, Saint-Aubin, France). For each exposure experiment, 250 000 cells per well within a 6-well collagen IV plate were used. In order to prevent primary cultured cells to deviate from the initial population, experiments were performed between the cell passages 4 and 8.

### Exposure system and experimental setup

**Figure 1:**
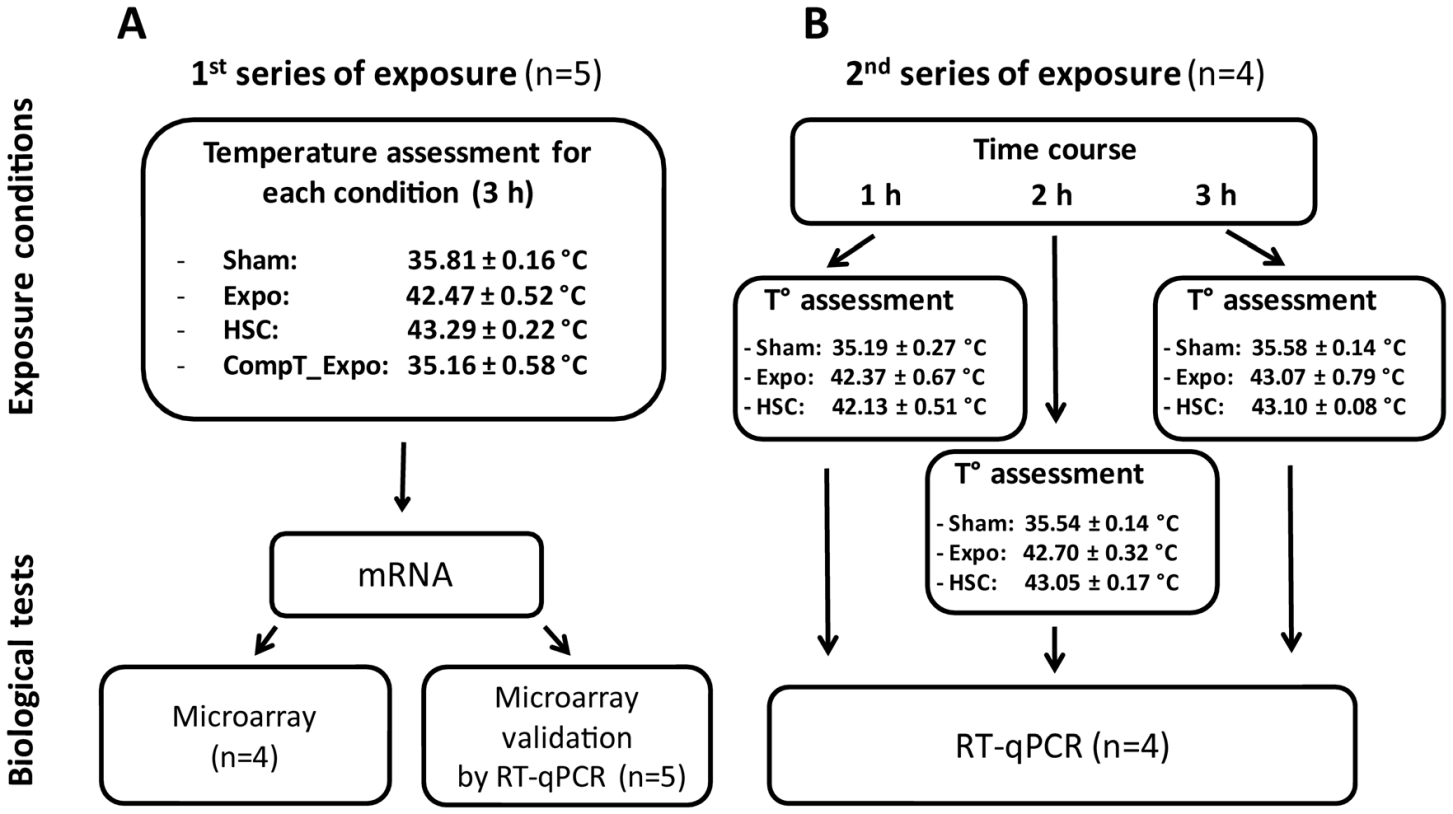
Experimental protocol. All experimental conditions are presented: 1) Control cells (Sham). 2) Cells exposed to MMW at 20 mW/cm^2^ (Expo). 3) Heat shocked cells (HSC). 4) Cells exposed to MMW with temperature increase compensated (CompT_Expo). Average of steady-state temperature in the medium is obtained from the measurement of each experimental replicates (°C, mean ± SD). A) First series of exposure with 4 conditions that were used for the microarray experiment and for the RT-PCR validation. B) Second series of exposure with 3 experimental conditions (n = 4) that are the duplication of the exposure, analysed by RT-PCR.

The MMW exposure system, allowing near-field exposure of cell cultures at 60 GHz, was previously described in detail [20]. Briefly, the exposure system consists of a signal generation sub-unit (QuinStar Technology Inc., CA, USA) containing a Gunn oscillator (center frequency f_c_ = 60.4 GHz), coupled to a frequency control sub-unit. The signal is amplified and transmitted to a 17 dB-gain standard pyramidal horn antenna placed in a MEMMERT UE400 incubator. The exposure conditions were optimized numerically to maximize the homogeneity of the SAR distribution within the cell monolayer using a standard horn antenna. Keratinocytes were exposed (Expo) as previously described [20] and control cells (Sham) were cultured under the same conditions, but with the MMW generator switched off. Cells were exposed during 3 hours at 60.4 GHz, with an average IPD of 20 mW/cm^2^. Average and peak SAR over the cell monolayer were equal to 594 W/kg and 1233 W/kg, respectively. Under these conditions, the exposure induces an increase of the culture medium temperature. In order to determine the importance of this heating effect on the cellular response, several controls were performed. The experimental protocol is described in Figure 1. Firstly, a heat shock control (HSC) was carried out by culturing unexposed cells at the same temperature as that obtained during the MMW exposure. Secondly, cells were exposed to MMW at the same steady-state temperature as observed in sham control. The temperature increase was compensated during the exposure by lowering the temperature of the cell incubator (CompT_Expo). During the experiments, the temperature was monitored using a 4-channel Reflex fiber optic thermometer (NEOPTIX, Quebec, Canada) (Figure 1A). We carried out five biological replicates for each experimental condition, and the order of the experiments was randomly performed.

To test the reproducibility and robustness of our data, a second series of exposure was carried out. All experiments were renewed for the validation of the candidate genes by RT-PCR. For this duplication, a time course was performed (1 to 3 hours of exposure, n = 4), and three experimental conditions (Sham, Expo and HSC) were followed. The temperatures were monitored as previously described (Figure 1B).

### RNA extraction

The RNA purifications were performed immediately after each exposure. RNAs were extracted with the Qiagen RNeasy kit (Qiagen, Hilden, Germany), and then quantified by Nanodrop 1000 spectrophotometer (Nanodrop technology, Cambridge, UK). All RNA extracts were analysed with bioanalyser apparatus and their RNA integrity Number (RIN) were between 9.8 and 10. RNAs were reverse-transcripted and the obtained cDNA were amplified and finally Cy3-labelled, according to supplier instructions (Agilent Technologies, Massy, France).

### Microarray

Gene expression profiling was carried out on Agilent Whole Human Genome 8×60K Microarrays (Agilent Technologies, Massy, France). Four independent RNA samples per condition (n = 4) were randomly analysed on one-color microarray. The complete data set was deposited in the Gene Expression Omnibus (GEO) database (http://www. ncbi.nlm.nih.gov/geo, GEO series accession number GSE57135). Data were log2-transformed and normalized (intra- and inter-array scaling). Microarray analysis was performed using GeneSpring GX software (Agilent Technologies, Massy, France). The number of positive probes was of 23767. Probes were then filtered on the basis of their expression level (intensity greater than 125 in at least one condition), and on their Standard Deviation (SD<0.5 in the two conditions compared). Genes differentially expressed between two conditions were identified by using Student's *t*-tests. Adjusted *p*-values were calculated by controlling for the false discovery rate (FDR), with the Benjamini & Hochberg (BH) correction for multiple testing. Genes were considered significantly differentially expressed if the adjusted *p*-value was below 0.05 and the absolute fold-change (FC) between conditions was above 2. Additional comparison was also performed considering FC>1.5 and adjusted *p*-value lower than 0.05. To complete the analysis, a hierarchical clustering was performed with the pooled gene entity lists obtained from Sham/Expo and Sham/HSC comparisons, using Pearson-correlation with the TIGR MultiExperiment Viewer Mev version 4.1 (Institute of Genomic Research, Rockville, MD).

### RT-PCR analysis

After microarray analysis, real time RT-PCR validation was performed on 22 genes entities that were selected from differentially expressed genes. The primers were designed by SA biosciences (Qiagen, Hilden, Germany) and all primers references are presented in Table S1 in File S1. Three housekeeping genes (*HPRT1*, *GAPDH* and *ACTB*) were used for normalization. The RT-PCRs were carried out from RNA samples of the first series of exposure (n = 5), and those of the second series (time course, n = 4). For all the experiments, 900 ng of RNA were reverse-transcripted with the RT^2^ first strand kit according to supplier instructions (Qiagen, Hilden, Germany), and analyzed onto Custom RT^2^ Profiler PCR array with RT^2^ SYBR green qPCR master mix onto MyIQ apparatus (Biorad, Hercules, CA). After real time RT-PCR assay, statistical analysis was performed using the one-tailed Wilcoxon - Mann Whitney test. The values are provided as the mean with their standard deviation (SD), and were considered statistically significant for *p*<0.05. Statistics were calculated with R from the BiostaTGV interface http://marne.u707.jussieu.fr/biostatgv/.

## Results

### Comparison between Sham and MMW exposed cells

**Figure 2:**
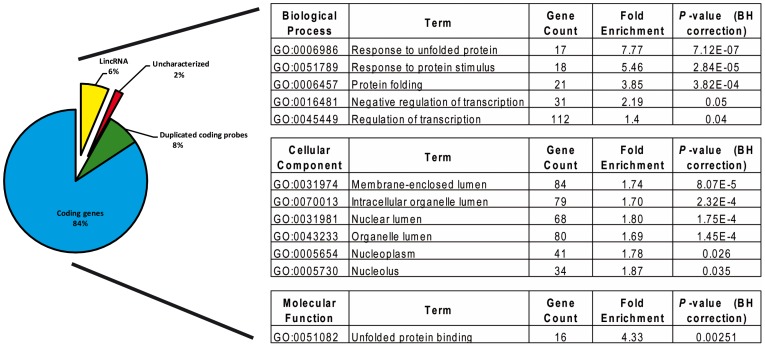
Classification of the 789 differentially expressed probes obtained after the comparison between MMW exposed cells (EXPO) and unexposed cells (Sham). Statistically enrichment of gene ontology (GO) of differentially expressed gene entities was obtained from David gene's functional annotation.

The global gene expression change between the 60-GHz exposed cells at 20 mW/cm^2^ (Expo) and unexposed cells (Sham) evidenced 789 differentially expressed probes with a fold change above 2 (Table 1 and Table S2 in File S1). This probe list included 665 annotated coding genes and 51 long intergenic non-coding RNAs (lincRNA). Among the coding genes, 366 (55%) were down-regulated (Expo <Sham) and 299 (45%) were up-regulated (Expo> Sham). It should be noted that when the microarray analysis is done with an absolute fold change filtered at 1.5, then 1172 probes were found to be differentially expressed (Table 1). Functional enrichments were performed using the DAVID software (David version 6.7; http://david.abcc.ncifcrf.gov/). Biological categories were considered enriched for a corrected *p*-value below 0.05. The main biological categories associated with these differentially expressed genes were chaperone and heat shock (Figure 2), which is consistent with the heat shock effect of MMW exposure in our conditions. Indeed, under this IPD exposure, the temperature in the cell medium is increased by 6.7°C (Figure 1). In fact, this differentially expressed gene list evidenced two main impacts of the MMW exposure on the cell (Figure 2). The first one is the response to unfolded protein that is a well-known consequence of the temperature increase. The second effect is the negative regulation of the gene expression that is illustrated by an increase in the expression of genes implicated in the negative regulation, and by the down regulation of most differentially expressed genes.

**Table 1:** Number of significantly differentially expressed probes between experimental conditions.

	Comparisons			
	FC cut-off	Sham vs Expo	Sham vs CompT_Expo	HSC vs Expo
Number of statistically differentially expressed probes	Absolute Fold Change ≥ 2	789	0	37
	Absolute Fold Change ≥ 1.5	1176	0	166

To verify whether MMW stimulation could initiate a global and long distance organism response, we focused on secreted factors. Among the differentially expressed genes, 26 (3.9%) were identified as coding for secreted proteins (Table 2), 15 were up-regulated and 11 were down-regulated. Seven of these secreted factors were associated with the functional category “response to wounding” that is merely linked with the increase of the temperature. No other GO terms were found to be statistically relevant.

**Table 2:** Secreted factors statistically differentially expressed between Sham and Expo.

Probe number	Gene symbol	Corrected *p*-value	Absolute Fold Change	Regulation	Gene name	Gene bank Accession number
**A_23_P215913**	**CLU**	0.0000451	20.64	up	clusterin	NM_203339
**A_23_P114626**	**SERPINC1**	0.000037	18.76	up	serpin peptidase inhibitor, clade C (antithrombin), member 1	NM_000488
**A_33_P3276693**	**PGF**	0.0000975	9.65	up	placental growth factor	NM_002632
**A_24_P268993**	**LEAP2**	0.000079	6.78	up	liver expressed antimicrobial peptide 2	NM_052971
**A_33_P3214948**	**SPOCK2**	0.000167	5.18	up	sparc/osteonectin, cwcv and kazal-like domains proteoglycan (testican) 2	NM_014767
**A_33_P3353030**	**UCN**	0.000337	3.63	up	urocortin	NM_003353
**A_33_P3262020**	**C8G**	0.00022	3.23	up	complement component 8, gamma polypeptide	NM_000606
**A_33_P3304668**	**COL1A1**	0.0018	3.2	up	collagen, type I, alpha 1	NM_000088
**A_24_P583040**	**C17orf67**	0.00031	2.72	up	chromosome 17 open reading frame 67	XM_001718395
**A_24_P110558**	**C5orf53**	0.00085	2.63	up	chromosome 5 open reading frame 53	NM_001007189
**A_23_P213319**	**ADAMTS6**	0.005	2.46	up	ADAM metallopeptidase with thrombospondin type 1 motif, 6	NM_197941
**A_23_P204630**	**NTN4**	0.0098	2.18	up	netrin 4	NM_021229
**A_33_P3286953**	**ADAMTS6**	0.0007	2.12	up	ADAM metallopeptidase with thrombospondin type 1 motif, 6	NM_197941
**A_23_P119916**	**WNT6**	0.00089	2.12	up	wingless-type MMTV integration site family, member 6	NM_006522
**A_23_P24129**	**DKK1**	0.0014	2.11	up	dickkopf homolog 1 (Xenopus laevis)	NM_012242
**A_24_P345846**	**ANTXR2**	0.0021	2.02	up	anthrax toxin receptor 2	NM_058172
**A_23_P127948**	**ADM**	0.0009	2.06	down	adrenomedullin	NM_001124
**A_23_P41344**	**EREG**	0.007	2.08	down	epiregulin	NM_001432
**A_23_P322**	**EFNA4**	0.002	2.11	down	ephrin-A4	NM_182690
**A_23_P53588**	**WNT5B**	0.0003	2.18	down	wingless-type MMTV integration site family, member 5B	NM_030775
**A_23_P150693**	**FJX1**	0.0025	2.23	down	four jointed box 1 (Drosophila)	NM_014344
**A_23_P162142**	**TSKU**	0.0019	2.29	down	tsukushin	NM_015516
**A_23_P380857**	**APOL4**	0.00078	2.41	down	apolipoprotein L, 4	NM_030643
**A_23_P212617**	**TFRC**	0.00018	2.53	down	transferrin receptor (p90, CD71)	NM_003234
**A_23_P424582**	**EGFL8**	0.000030	2.55	down	EGF-like-domain, multiple 8	NM_030652
**A_23_P2271**	**PTHLH**	0.0000935	2.68	down	parathyroid hormone-like hormone	NM_198965
**A_23_P110531**	**FST**	0.00057	3.8	down	follistatin	NM_013409

### Differential gene expression between unexposed cells and 60-GHz exposed cells with a compensation of the temperature increase (Sham vs CompT_Expo)

In order to dissociate the thermal from the pure electromagnetic effects of MMW, the temperature increase induced by exposure was compensated by decreasing the incubator temperature (CompT_Expo). Under these conditions, Sham and CompT_Expo cells were cultivated at the same physiological temperature, but with only the MMW exposure status changing. The medium temperature was assessed for each experiment and was similar for both conditions (Figure 1). The whole gene expression under this exposure scenario was then compared to unexposed cell (Sham). No gene was found significantly differentially expressed between these two conditions (Table 1). Therefore, without a temperature rise, acute MMW exposure did not modify the whole gene expression of keratinocytes primary cultures.

### Differential gene expression between unexposed cells and heat shock control (Sham vs HSC)

As exposure at 20 mW/cm^2^ induces a heat shock (Figure 1), we performed a heat shock control (HSC). While comparing the HSC and the unexposed Sham condition, we evidenced 1018 differentially expressed probes with a fold change above 2 and BH corrected *p*-value<0.05. This probe list included 811 annotated unique coding genes and 76 long intergenic non-coding RNAs (lincRNA). Remaining probes consisted in unannotated or hypothetical genes. Functional enrichments were performed using the DAVID software. Biological categories were considered enriched for a corrected *p*-value below 0.05. The main biological categories associated with up-regulated genes were stress response, chaperone, molecular chaperone and heat shock, which is consistent with the heat shock condition. For the down-regulated genes, the main target functions are transcription, regulation of the transcription and DNA-binding protein. It is interesting to note that these functional categories are the same as those which had been found when Sham and MMW-exposed cells were compared. For example, the gene ontology of the top 100 entities up-regulated in the HSC and Expo, both correspond to molecular function associated to unfold protein binding or heat shock protein binding. Considering the overall genes differentially expressed, we performed a Spearman's rank correlation rho on the global fold-change observed between HSC and Expo versus Sham control. The rho observed were 0.8 (*p-*value of 2.2.10^−16^). That evidenced a significant correlation between heat effect and MMW effect on the gene expression. The hierarchical clustering of the down-regulated (Figure S1A) genes and the up-regulated genes (Figure S1B) showed that the expression profiles are similar. Some dissimilarity was observed and should be nevertheless linked to heat generation method that could be quite different even if the final temperatures between conditions are the same. In the hierarchical clustering of the up-regulated genes a specific cluster was nevertheless isolated (indicated by a bar in Figure S1B). This original cluster evidences up-regulated factors in Expo condition but not in HSC condition (Figure S1C). This cluster contains 7 genes (*DKK1, NTN4, ADCY7, IRF2BP2, ADAMTS6, FAM120C and PAG1*). Altogether these data suggest that the main effect of MMW is linked to the heat shock associated effect, except for some genes that may illustrate an action of MMW independent from the heat.

### Differential gene expression between 60-GHz exposition and similar heat shock control (Expo vs HSC)

To confirm this observation, the whole gene expression between Expo and HSC was compared. Thirty seven probes were found statistically (*p*<0.05) differentially expressed with a FC>2 (Table 1 and Table S3 in File S1). Among these probes, 34 corresponded to coding genes and 3 to non-coding RNA (1 small nucleolar RNA, 2 long intergenic non coding RNA). All 34 genes were up-regulated between HSC and Expo (Expo> HSC), and only 3 (*ADAMTS6, NOG* and *IL7R*) represent secreted factors. The same analysis was performed with a cut-off value of 1.5 fold induction: 166 probes were found differentially expressed, with 13 genes down regulated and 153 up-regulated (Table S3 in File S1). It should be noted that in this list, we found 4 of the 7 genes previously identified in the specific cluster (*DKK1, IRF2BP2, ADAMTS6, FAM120C*). The 3 remaining genes (*NTN4, ADCY7* and *PAG1)* were excluded by the statistical test, because their expression presents a strong variability in heat shock condition (Figure S1C).

### Quantitative RT-PCR validation, duplication of the experiments and time course of the gene expression

Twenty-two genes were selected on the basis of their fold change and *p*-value. Fifteen had an absolute FC higher than 2, and 7 had a FC between 1.5 and 2. All selected genes are detailed in Table 3. A first RT-PCR validation step was performed with the mRNA used for microarray, plus one additional replicate for each tested conditions (Sham, Expo and HSC). 21 genes out of 22 were confirmed as differentially expressed (Table 3).

**Table 3:** Selected genes for RT-PCR validation.

	Microarray (n = 4)		RT-PCR (n = 5)		RT-PCR 3h-validation (n = 4)	
Gene Entity	FC	*t-*test (BH) *p*-value	FC	MW test *p*-value	FC	MW test *p-*value
**NOG**	4.43	0.028	4.93	0.00395	2.39	0.171
**HIST1H1A**	2.92	0.028	2.93	0.00395	2.22	0.1
**JUNB**	2.74	0.028	3.84	0.00395	3.14	0.014
**MYC**	2.74	0.028	2.53	0.00395	1.55	0.343
**SEMA4C**	2.58	0.028	3.14	0.008	1.87	0.057
**SIK1**	2.39	0.028	3.05	0.00395	1.30	0.343
**FADD**	2.25	0.028	2.12	0.00395	2.17	0.014
**IL7R**	2.2	0.032	1.63	0.0475	2.34	0.057
**POLR1C**	2.15	0.029	1.71	0.00395	1.17	0.171
**PHF13**	2.12	0.029	2.14	0.00395	1.18	0.5
**FOXL2**	2.11	0.029	1.73	0.0475	1.0	0.5
**ADAMTS6**	2.07	0.028	1.78	0.00395	2.03	0.029
**C14orf169**	2.04	0.028	1.96	0.00395	1.74	0.057
**SNAI2**	2.04	0.029	1.78	0.008	1.29	0.443
**FANCF**	2.03	0.032	1.77	0.00395	1.26	0.24
**ZFP36L1**	2.03	0.028	1.94	0.00395	1.57	0.171
**SOX4**	2.00	0.034	2.65	0.00395	1.12	0.443
**TGIF1**	1.96	0.028	2.09	0.00395	1.23	0.343
**JAG1**	1.95	0.028	1.95	0.00395	1.16	0.443
**RASSF1**	1.89	0.028	2.96	0.00395	1.74	0.057
**NEDD4L**	1.8	0.032	1.57	0.00395	1.09	0.5
**KCTD12**	1.71	0.044	1.60	0.075	1.06	0.443

List of genes determined as differentially expressed between Expo and HSC. The table includes the fold change (FC) and the *p*-value from microarray experiments, first and second RT-PCR validations (duplication of the exposure, after 3 hours of exposure, IPD = 20 mW/cm^2^). BH: Benjamini and Hochberg correction for multiple testing; MW: Wilcoxon - Mann Whitney test.

**Figure 3:**
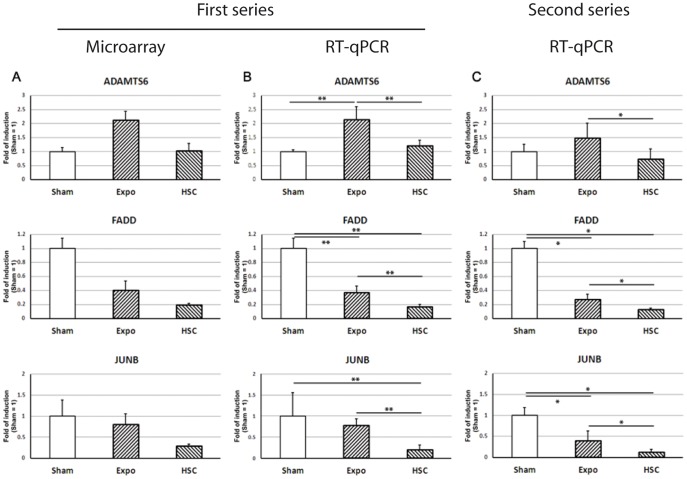
Expression profiles of the 3 validated genes differentially expressed after 3 h of exposure. A) Microarray gene profile, B) RT-PCR validation with the same RNA extracts, C) RT-qPCR validation by the whole exposure duplication (3 hours of treatments). * *p*-value<0.05, ** *p*-value<0.01 from one tailed Wilcoxon-Mann-Whitney *U*-test.

**Figure 4:**
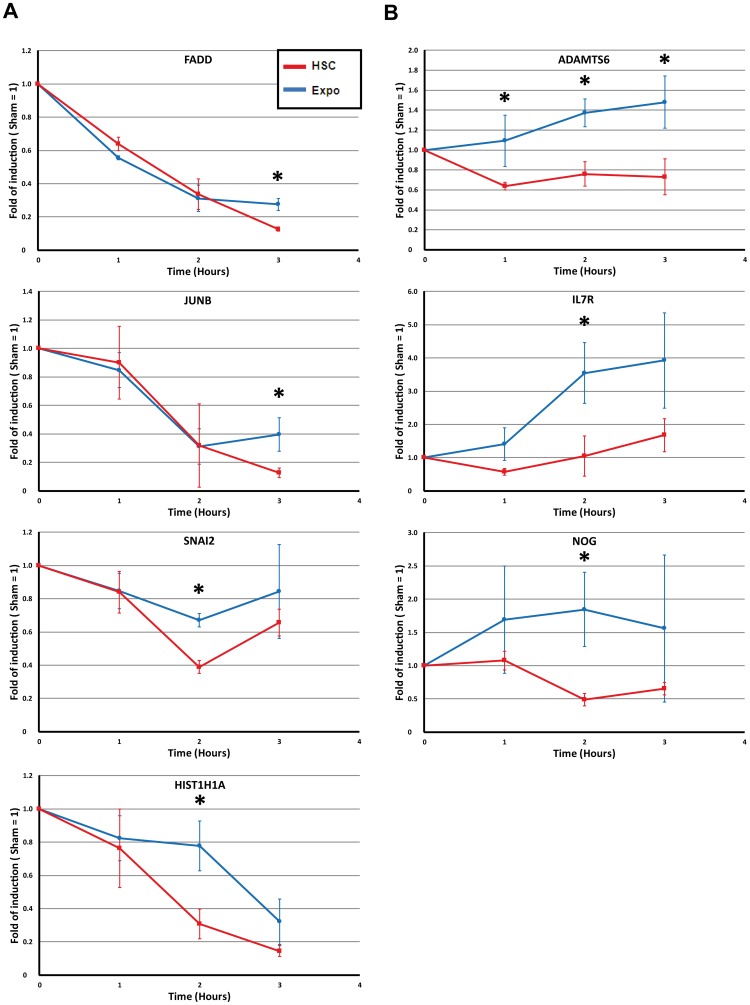
Time course profiles of the validated differentially expressed genes entities. A) Temperature dependent regulated genes that evidence a MMW impact on kinetic profiles, B) Specifically MMW up-regulated genes entities. Each point corresponds to the average +/− SEM. Sham unexposed point is normalised to 1 for each point of the kinetics. * *p*-value<0.05 from one tailed Wilcoxon-Mann-Whitney *U*-test.

 In order to verify the reproducibility of the observed MMW effect on the gene expression, a second series of exposures was performed. Thus, time course experiments were done to determine the dynamic of the 22 genes expression during the exposure (Table 3, Figures 3 and 4). After this second series of exposures and RT-PCR experimentations, only 7 genes were confirmed as differentially expressed. These genes are: *ADAM metallopeptidase with thrombospondin type 1 motif 6* (*ADAMTS6*), *FAS (TNFRSF6)-Associated via Death Domain* (*FADD*), *Jun B Proto-Oncogene* (*JUNB*), *Noggin* (*NOG*), *Interleukin 7 receptor* (*IL7R*), *Snail family zinc finger 2* (*SNAI2*) and *Histone cluster 1, H1a* (*HIST1H1A*). It is interesting to note that among these genes, only 3 have shown a differential expression after 3 hours of exposure: *ADAMTS6, FADD and JUNB* (Figure 3). Four genes (*IL7R, NOG, SNAI2* and *HIST1H1A*) were statistically validated only after 2 hours of exposure and the expression profiles of these genes remained equivalent but not significant at 3 hours (Figure 4). According to these time-course profiles, two main groups could be delineated. The first one contained the majority of the genes. Compared to the sham control, the expression of these genes decreased during both Expo and HSC conditions (Figure 4A). It is important to note that the differences between HSC and EXPO are very weak and on the verge of the significance of the statistical test. Those profiles clearly show a same trend in the transcriptional response. This may illustrate the heat effect of the MMW exposure, with a slight difference in heat generation between Expo and HSC, even if we cannot exclude the existence of false positives. The second group (3 genes: *ADAMTS6, NOG* and *IL7R*) showed an increase in gene expression under MMW exposure and a slight decrease in HSC expression (Figure 4B). This group is interesting because it seemed to evidence a specific effect of MMW on gene expression that was different from both HSC and sham conditions.

## Discussion

This study aimed to evaluate whether acute high intensity exposure to MMW could deeply modify the whole gene expression of keratinocytes. The originality of our work is that we exposed cells at an IPD equivalent to the limit of the ICNIRP guidelines, and that *in vitro* studies constitute a worst-case model because increase of the temperature could not be limited by the blood flow, like in *in vivo* models. We evaluated the global impact of MMWs associated with the normal increase of temperature due to the heat effect at 20 mW/cm^2^. Under this condition, the microarray experiments identified 665 genes differentially expressed between the sham and exposed cells. We used gene ontology (GO) classification to identify which critical pathways and processes are linked with these genes. Many of these genes are associated with the response of cellular stress, such as heat-shock protein genes. This clearly indicates that the effect of high-power MMW exposure on gene expression is due in part to hyperthermia, which confirms previous observation made at lower MMW frequency at 35 GHz, 75 mW/cm^2^ [26]. In our experimental design, the high number of modified genes (665) shows that the ICNIRP current limit is probably too permissive to prevent biological response. However, this ICNIRP general public standard is only permitted for a very limited exposure area (1 cm^2^). It doubtless restricts the impact of the MMW exposure at the level of the whole body, but raises the question of the initiation of short or long distance organism response by secreted factors. Over-expression of secreted factors may explain the therapeutic effects of MMW on the immune or neural systems. We found 26 secreted factors (Table 2), and among them 15 were up-regulated. The GO-term associated to these genes was mainly linked to cellular differentiation (data not shown), and only few genes were related to immune or paracrine factors. However, it should be noted that our study focused on transcripts and it will be important in the future to determine whether these changes in gene expression could also be detected at the protein level.

To identify the effect of the MMW radiation, independently from the heat shock effect, different controls were performed. First, keratinocytes were exposed to MMW at constant physiological temperature. Therefore, our study shows that when temperature is artificially maintained at 35°C, the 60-GHz radiations did not significantly modify the transcriptome of keratinocytes. These results are in agreement with our previously published data [25] and with data obtained at another frequency without an increase of temperature [27]. Then, to determine the role of the temperature rise, a heat shock control (HSC) was performed under the same condition of culture and temperature. When this HSC was compared to the MMW exposure, 34 genes were found as differentially expressed (FC<2). This result indicates that cell heating could not perfectly mimic the cellular response induced by MMW. The exposure duplication and the RT-PCR validation have evidenced that 7 genes are differentially expressed; indicating that MMW radiation slightly but significantly modifies the gene expression adaptation of cells submitted to a heat stress. Two hypotheses could explain this effect: 1) at the cellular level, the heat generation method is not totally analog. In HSC the heat comes by convection, while MMW exposure increases the water molecule rotation; 2) MMW could slightly modify a signaling pathway relative to heat shock response. With regard to this last hypothesis, 3 genes show an interesting expression profile. *ADAMTS6*, *NOG* and *IL7R* show a specific MMW-induced expression, when heat and MMW are combined. These results suggest that a peculiar pathway has been activated by exposure. This signaling pathway remains to be characterized and could be useful for the comprehension of the possible interaction between MMWs and living cells.

The main intriguing part is that these three up-regulated genes belong to the secreted factors and are therefore implicated in the cellular communication. *IL7R* is mainly implicated in the normal B- and T-cell development [28], [29]. *IL7R* exists on two main splice variants: a membrane included *IL7R* that is implicated in the IL7 signaling transduction and a secreted form whose function remains to be determined. This gene is implicated in several pathologies such as leukemia [28], [29], autoimmunity [30], and arthritis [31]. In case of rheumatoid arthritis both, membrane and soluble IL7R, are induced by pro-inflammatory cytokine, especially TNFα [32]. *ADAMTS6* belongs to the desintegrin and metalloproteinase with thrombospondin domain. Even if the function of this secreted protease is currently unknown, it seems to be linked with the extracellular matrix degradation and inflammatory. This gene seems to be implicated in osteoarthritis and pain [33], intervertebral disc aging [34], as well as in colorectal cancer [35]. This secreted factor is also under the control of TNFα [36]. *Noggin* (*NOG*) is implicated in several steps of the human and animal development. NOG is a secreted factor that inhibits the bone morphogenetic protein (BMP), which creates a gradient, crucial for the development of embryo [37]. It is interesting to note that *noggin* expression is under the control of TGFβ, IL17, IFNγ and TNFα which induces an increase in noggin secretion [38]. To summarize, we can highlight the fact that these 3 secreted factors are linked to usual pathologies treated by the MMW. Moreover, their expressions are all three regulated by TNFα, and the TNFα/IFNγ signalization pathways were already suspected to be involved in therapeutic effects of MMW [14], [15], [39]. This opens a possible avenue for future research into the mechanisms of MMW interactions at molecular and cellular levels. Moreover, as the main identified genes are secreted factors involved in the cellular communication, the long-term or chronic cellular effects (which were not evaluated in this publication) should be performed in the future. It will give better idea of the real risk induced by MMW exposure, as up to now no data are available.

## Conclusion

In this study we aimed to assess whether MMW exposure could modify keratinocyte whole gene expression with an IPD at the limit of the general public ICNIRP recommendation. As MMW exposure has two components (electromagnetic and thermal), we introduced adequate heat control in order to dissociate thermal from pure electromagnetic effects. High-power MMW exposure induces a drastic modification of the whole gene expression that is mainly associated with the MMW's thermal effect. However, heat control did not mimic exactly the whole gene expression modification, and we found 7 genes that were significantly differentially expressed. Among them, 3 genes (*ADAMTS6*, *NOG* and *IL7R*) encode secreted factors that are specifically MMW-induced. Thus, we evidence here for the first time, that acute MMW stimulation specifically induces the expression of some secreted genes. Additional studies will be needed to determine what are the molecular mechanisms underlying this cellular response and how could evolve this response after chronic long-term MMW exposure.

## Supporting Information

Figure S1 **Hierarchical Clustering of the Microarray Data.** Three culture conditions were tested (n = 4 each): Exposed to millimeter waves (Expo), heat shock control (HSC) and control cells (Sham). Heatmap of the significantly expressed probes. Each row represents an individual gene entity, and each column represents an individual RNA sample. Expression levels of gene entities are symbolized by a code color: red indicates highest expression and green indicates lowest expression. A) Hierarchical clustering for genes down-regulated under HSC and MMW exposure versus Sham control. B) Hierarchical clustering for genes up-regulated under HSC and MMW exposure. The bar indicates a distinct cluster presented in C. C) Distinct cluster which includes up-regulated genes in Expo condition but not in HSC condition.
(TIF)

File S1 **Supporting tables.** Table S1, List of primer used for RT-qPCR experiment designed from SA bioscience. Table S2, List of the 789 diffentially expressed probes obtained from *t*-test with BH correction between EXPO and Sham (up: Expo>sham). Table S3, List of the 166 diffentially expressed probes obtained from *t*-test with BH correction between EXPO and HSC (up: EXPO> HSC).
(DOC)
