# Supplementary figures and images for: Transcriptome Analysis Reveals the Contribution of Thermal and the Specific Effects in Cellular Response to Millimeter Wave Exposure
Source: PLoS One. 2014 Oct 10;9(10):e109435. doi: 10.1371/journal.pone.0109435 (PMC4193780; doi:10.1371/journal.pone.0109435)

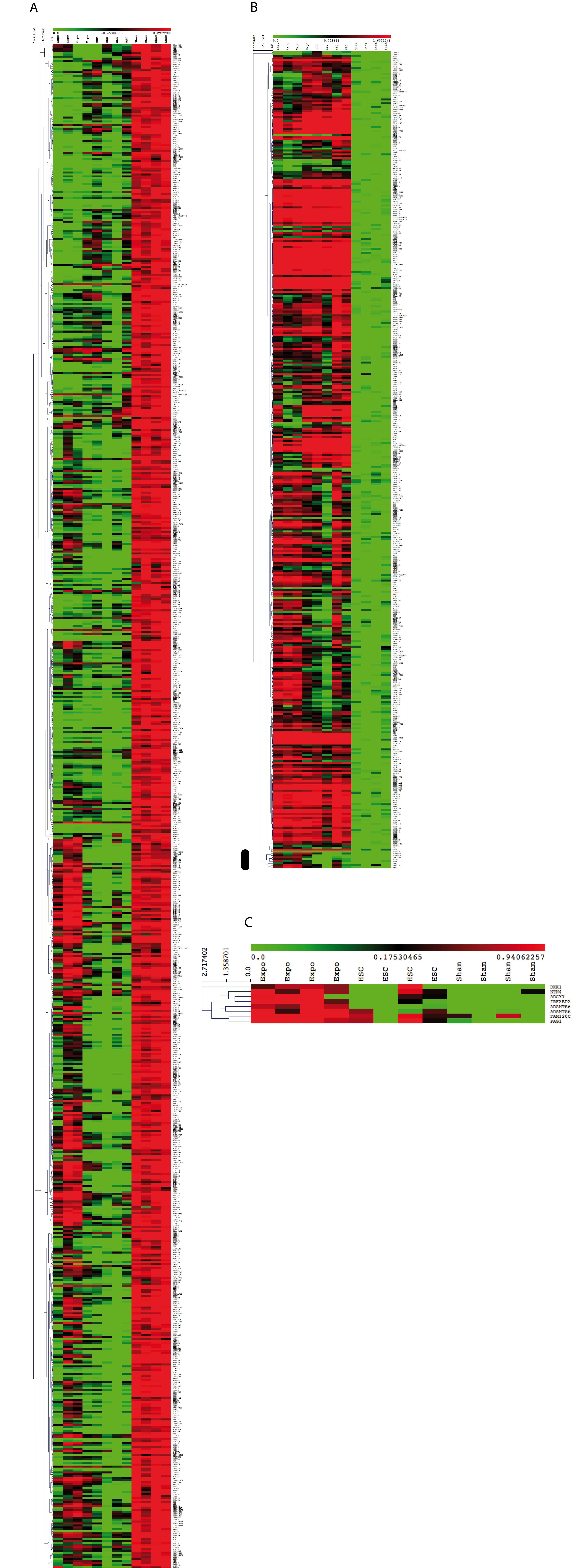

Supplement: Figure S1 — Hierarchical Clustering of the Microarray Data. Three culture conditions were tested (n = 4 each): Exposed to millimeter waves (Expo), heat shock control (HSC) and control cells (Sham). Heatmap of the significantly expressed probes. Each row represents an individual gene entity, and each column represents an individual RNA sample. Expression levels of gene entities are symbolized by a code color: red indicates highest expression and green indicates lowest expression. A) Hierarchical clustering for genes down-regulated under HSC and MMW exposure versus Sham control. B) Hierarchical clustering for genes up-regulated under HSC and MMW exposure. The bar indicates a distinct cluster presented in C. C) Distinct cluster which includes up-regulated genes in Expo condition but not in HSC condition. (TIF) [file pone.0109435.s001.tif]
