# Supplementary material for: Transcriptome Analysis Reveals the Contribution of Thermal and the Specific Effects in Cellular Response to Millimeter Wave Exposure
Source: PLoS One. 2014 Oct 10;9(10):e109435. doi: 10.1371/journal.pone.0109435 (PMC4193780; doi:10.1371/journal.pone.0109435)
Supplement: File S1 — Supporting tables. Table S1, List of primer used for RT-qPCR experiment designed from SA bioscience. Table S2, List of the 789 diffentially expressed probes obtained from t-test with BH correction between EXPO and Sham (up: Expo>sham). Table S3, List of the 166 diffentially expressed probes obtained from t-test with BH correction between EXPO and HSC (up: EXPO> HSC). (DOC) [file pone.0109435.s002.doc]

**FILE S1: SUPPLEMENTAL MATERIAL**

**Transcriptome analysis reveals the contribution of thermal and the specific effects in cellular response to millimeter wave exposure**

Denis Habauzit, Catherine Le Quément, Maxim Zhadobov, Catherine Martin, Marc Aubry, Ronan Sauleau and Yves Le Dréan.

**Table of contents**

# Experimental design of microarrays……………………………………………………………………………………………………….… 2

# Legend of Figure S1…………………………………………………………………………………………………………………………… 4

# Table S1. List of primer used for RT-qPCR experiment designed from SA bioscience………….………………………………………. 5

# Table S2. List of the 789 diffentially expressed probes obtained from *t*-test with BH correction between Sham and EXPO………… 6

# Table S3. List of the 166 diffentially expressed probes obtained from *t*-test with BH correction between EXPO and HSC…………. 35

**Experimental design of microarrays.**

Primary human keratinocytes were cultured (250 000 cells per well) within a 6-well collagen IV plate (Becton Dikinson Franklin Lakes, NJ), in supplemented Keratinocyte-SFM medium (Gibco, Carlsbad, CA) with antibiotics (Invitrogen, Saint-Aubin, France).

Four experimental conditions were used: 1) EXPO: keratinocytes were exposed during 3 hours at 60.4 GHz, with an average IPD of 20 mW/cm². 2) Sham: cells were cultured under the same conditions, but the MMW generator switched off. 3) HSC: heat shock control corresponding to unexposed cells cultivated at the same temperature as that obtained during the MMW exposure (43°C). 4) CompT_Expo: cells were exposed to MMW at the same steady-state temperature as observed in sham control (35°C).

Four independent biological replicates per condition were used for microarray analysis

Immediately after exposure, RNA was prepared using the RNAeasy column purification (Qiagen, Hilden, Germany), following the manufacturer's recommendations. RNA was quantified using a NanoDrop-1000 spectrophotometer (Nanodrop technology, Cambridge, UK) and quality was monitored with the Agilent 2100 Bioanalyzer (Agilent Technologies, Santa Clara, CA) and the overall RNA integrity numbers (RIN) were between 9.8 and 10.

Cyanine-3 (Cy3) labeled cRNA was prepared from 0.1 µg RNA using the LowInput QuickAmp Labeling Kit One-Color (Agilent) according to the manufacturer's instructions, followed by RNAeasy column purification (QIAGEN, Valencia, CA). Dye incorporation and cRNA yield were checked with the NanoDrop ND-1000 Spectrophotometer.

0.6 µg of Cy3-labelled cRNA was fragmented at 60°C for 30 minutes in a reaction volume of 25 µl containing 1x Agilent fragmentation buffer and 2x Agilent blocking agent following the manufacturer’s instructions. On completion of the fragmentation reaction, 25 µl of 2x Agilent hybridization buffer was added to the fragmentation mixture and hybridized to Agilent Whole Human Genome Oligo Microarrays (8×60K) for 17 hours, at 65°C in a rotating Agilent hybridization oven. After hybridization, microarrays were washed 1 minute at room temperature with GE Wash Buffer 1 (Agilent) and 1 minute with GE Wash buffer 2 (Agilent) pre-warmed at 37°C. Microarrays were then dried immediately by brief centrifugation.

Slides were scanned immediately after washing on the Agilent DNA Microarray Scanner (G2505C) using one color scan setting for 8x60k array slides.

The scanned images were analyzed with Feature Extraction Software 9.1 (Agilent) using default parameters (protocol GE1_107 and Grid: 028004_D_F_20101102) to obtain background subtracted and spatially detrended Processed Signal intensities. Features flagged in Feature Extraction as Feature Non-uniform outliers were excluded.

Data were log2-transformed and then intra- and inter-array scaling were performed to normalize the signal intensities (quantile normalization and baseline transformation) by using GeneSpring GX software (Agilent Technologies).

**Figure S1. Hierarchical Clustering of the Microarray Data**

Three culture conditions were tested (n=4 each): Exposed to millimeter waves (Expo)), heat shock control (HSC) and control cells (Sham). Heatmap of the significantly expressed probes. Each row represents an individual gene entity, and each column represents an individual RNA sample. Expression levels of gene entities are symbolized by a code color: red indicates highest expression and green indicates lowest expression. A) Hierarchical clustering for genes down-regulated under HSC and MMW exposure versus Sham control. B) Hierarchical clustering for genes up-regulated under HSC and MMW exposure. The bar indicates a distinct cluster presented in C. C) Distinct cluster which includes up-regulated genes in Expo condition but not in HSC condition.

Table S1. List of primer used for RT-qPCR experiment designed from SA bioscience

| **Gene Symbol** | **Alias** | **Refseq #** | **Official Full Name** | **RT2 Catalog Number** |
| --- | --- | --- | --- | --- |
| ADAMTS6 | ADAM-TS 6/ADAM-TS6/ADAMTS-6 | NM_197941 | ADAM metallopeptidase with thrombospondin type 1 motif, 6 | PPH15788 |
| C14orf169 | FLJ21802/MAPJD/NO66 | NM_024644 | Chromosome 14 open reading frame 169 | PPH09367 |
| FADD | MGC8528/MORT1 | NM_003824 | Fas (TNFRSF6)-associated via death domain | PPH00367 |
| FANCF | FAF/MGC126856 | NM_022725 | Fanconi anemia, complementation group F | PPH09749 |
| FOXL2 | BPES/BPES1/PFRK/PINTO/POF3 | NM_023067 | Forkhead box L2 | PPH01976 |
| HIST1H1A | H1.1/H1A/H1F1/HIST1/MGC126642/MGC138345 | NM_005325 | Histone cluster 1, H1a | PPH12777 |
| IL7R | CD127/CDW127/IL-7R-alpha/IL7RA/ILRA | NM_002185 | Interleukin 7 receptor | PPH00607 |
| JAG1 | AGS/AHD/AWS/CD339/HJ1/JAGL1/MGC104644 | NM_000214 | Jagged 1 | PPH06022 |
| JUNB | AP-1 | NM_002229 | Jun B proto-oncogene | PPH00179 |
| KCTD12 | C13orf2/FLJ33073/KIAA1778/PFET1/PFETIN | NM_138444 | Potassium channel tetramerisation domain containing 12 | PPH11116 |
| MYC | MRTL/bHLHe39/c-Myc | NM_002467 | V-myc myelocytomatosis viral oncogene homolog (avian) | PPH00100 |
| NEDD4L | FLJ33870/KIAA0439/NEDD4-2/RSP5/hNedd4-2 | NM_015277 | Neural precursor cell expressed, developmentally down-regulated 4-like | PPH15046 |
| NOG | SYM1/SYNS1 | NM_005450 | Noggin | PPH01926 |
| PHF13 | MGC43399/PHF5/SPOC1 | NM_153812 | PHD finger protein 13 | PPH07499 |
| POLR1C | RPA39/RPA40/RPA5/RPAC1/TCS3 | NM_004875 | Polymerase (RNA) I polypeptide C, 30kDa | PPH01762 |
| RASSF1 | 123F2/NORE2A/RASSF1A/RDA32/REH3P21 | NM_007182 | Ras association (RalGDS/AF-6) domain family member 1 | PPH08711 |
| SEMA4C | FLJ20369/KIAA1739/M-SEMA-F/MGC126382/MGC126383/SEMACL1/SEMAF/SEMAI | NM_017789 | Sema domain, immunoglobulin domain (Ig), transmembrane domain (TM) and short cytoplasmic domain, (semaphorin) 4C | PPH07485 |
| SIK1 | MSK/SIK/SNF1LK | NM_173354 | Salt-inducible kinase 1 | PPH18631 |
| SNAI2 | MGC10182/SLUG/SLUGH1/SNAIL2/WS2D | NM_003068 | Snail homolog 2 (Drosophila) | PPH02475 |
| SOX4 | EVI16 | NM_003107 | SRY (sex determining region Y)-box 4 | PPH01950 |
| TGIF1 | HPE4/MGC39747/MGC5066/TGIF | NM_003244 | TGFB-induced factor homeobox 1 | PPH01953 |
| ZFP36L1 | BRF1/Berg36/ERF-1/ERF1/RNF162B/TIS11B/cMG1 | NM_004926 | Zinc finger protein 36, C3H type-like 1 | PPH10317 |

# Table S2. List of the 789 diffentially expressed probes obtained from *t*-test with BH correction between EXPO and Sham (up: Expo>sham).

| ProbeName | p-value | FC Absolute | Regulation  up: Expo>sham | GeneSymbol | Genbank Accession | GeneName |
| --- | --- | --- | --- | --- | --- | --- |
| A_23_P70547 | 9.85E-08 | 81.18657 | up | HSPA1L | NM_005527 | heat shock 70kDa protein 1-like |
| A_23_P206140 | 9.76E-07 | 78.65842 | up | DNAJA4 | NM_018602 | DnaJ (Hsp40) homolog. subfamily A. member 4 |
| A_33_P3415430 | 1.59E-08 | 54.082523 | up | HSPA1B | NM_005346 | heat shock 70kDa protein 1B |
| A_23_P111132 | 5.44E-09 | 46.12103 | up | HSPA1A | NM_005345 | heat shock 70kDa protein 1A |
| A_24_P350437 | 1.59E-06 | 33.21681 | up | THAP2 | NM_031435 | THAP domain containing. apoptosis associated protein 2 |
| A_23_P215913 | 1.44E-06 | 20.64114 | up | CLU | NM_203339 | clusterin |
| A_23_P90062 | 5.80E-06 | 19.285849 | up | DNAJB1 | NM_006145 | DnaJ (Hsp40) homolog. subfamily B. member 1 |
| A_23_P114626 | 7.20E-07 | 18.755402 | up | SERPINC1 | NM_000488 | serpin peptidase inhibitor. clade C (antithrombin). member 1 |
| A_23_P47077 | 6.11E-09 | 17.321936 | up | BAG3 | NM_004281 | BCL2-associated athanogene 3 |
| A_33_P3348752 | 1.36E-06 | 16.772524 | up | HSPH1 | NM_006644 | heat shock 105kDa/110kDa protein 1 |
| A_23_P88119 | 7.52E-08 | 14.625582 | up | HSPH1 | NM_006644 | heat shock 105kDa/110kDa protein 1 |
| A_24_P207479 | 2.24E-07 | 11.482053 | up | DEDD2 | NM_133328 | death effector domain containing 2 |
| A_33_P3340752 | 3.17E-06 | 10.167025 | up |  |  |  |
| A_33_P3277110 | 2.37E-06 | 9.928327 | up | SLC5A3 | NM_006933 | solute carrier family 5 (sodium/myo-inositol cotransporter). member 3 |
| A_23_P426305 | 7.34E-06 | 9.802168 | up | AOC3 | NM_003734 | amine oxidase. copper containing 3 (vascular adhesion protein 1) |
| A_33_P3276693 | 8.66E-06 | 9.651531 | up | PGF | NM_002632 | placental growth factor |
| A_23_P34396 | 8.24E-09 | 9.575392 | up | C1orf63 | NM_020317 | chromosome 1 open reading frame 63 |
| A_33_P3271387 | 6.35E-07 | 9.235936 | up | THAP3 | NM_138350 | THAP domain containing. apoptosis associated protein 3 |
| A_19_P00331981 | 1.37E-06 | 9.045805 | up |  |  |  |
| A_33_P3324186 | 1.21E-07 | 8.425033 | up | LOC642366 | CR936786 | hypothetical LOC642366 |
| A_23_P171237 | 2.69E-06 | 8.155941 | up | ACRC | NM_052957 | acidic repeat containing |
| A_33_P3502037 | 1.23E-06 | 7.9966984 | up | CHORDC1 | NM_012124 | cysteine and histidine-rich domain (CHORD)-containing 1 |
| A_19_P00809471 | 4.34E-09 | 7.904323 | up |  |  |  |
| A_32_P224850 | 2.67E-07 | 7.634831 | up | LOC645955 | XM_933296 | hypothetical LOC645955 |
| A_24_P9671 | 1.13E-07 | 7.303677 | up | DNAJA1 | NM_001539 | DnaJ (Hsp40) homolog. subfamily A. member 1 |
| A_23_P304511 | 5.67E-06 | 7.199554 | up | ZNF397 | NM_032347 | zinc finger protein 397 |
| A_33_P3807268 | 1.61E-08 | 7.003049 | up | HEJ1 | AF395440 | similar to DNAJ |
| A_23_P200801 | 5.02E-06 | 7.001208 | up | PDE4DIP | NM_001002811 | phosphodiesterase 4D interacting protein |
| A_19_P00323103 | 6.43E-06 | 6.8609624 | up |  |  |  |
| A_24_P268993 | 5.69E-06 | 6.7801547 | up | LEAP2 | NM_052971 | liver expressed antimicrobial peptide 2 |
| A_23_P74467 | 1.67E-05 | 6.673123 | up | KIAA0907 | NM_014949 | KIAA0907 |
| A_23_P99226 | 3.32E-05 | 6.5832286 | up | SIRT4 | NM_012240 | sirtuin (silent mating type information regulation 2 homolog) 4 (S. cerevisiae) |
| A_32_P204205 | 3.59E-06 | 6.1871696 | up | SIX4 | NM_017420 | SIX homeobox 4 |
| A_23_P336992 | 4.06E-06 | 6.113383 | up | ZFAND2A | NM_182491 | zinc finger. AN1-type domain 2A |
| A_24_P393958 | 9.47E-05 | 5.933411 | up | DNAJB4 | NM_007034 | DnaJ (Hsp40) homolog. subfamily B. member 4 |
| A_33_P3214343 | 4.02E-05 | 5.9325895 | up | PLCXD2 | NM_001134478 | phosphatidylinositol-specific phospholipase C. X domain containing 2 |
| A_23_P152583 | 4.75E-05 | 5.8992043 | up | ENGASE | NM_001042573 | endo-beta-N-acetylglucosaminidase |
| A_23_P384532 | 8.27E-05 | 5.873574 | up | CCDC11 | NM_145020 | coiled-coil domain containing 11 |
| A_23_P138805 | 1.93E-06 | 5.863304 | up | CHORDC1 | NM_012124 | cysteine and histidine-rich domain (CHORD)-containing 1 |
| A_23_P387471 | 3.51E-06 | 5.8550625 | up | MICB | NM_005931 | MHC class I polypeptide-related sequence B |
| A_19_P00331034 | 9.49E-06 | 5.744266 | up |  |  |  |
| A_33_P3685572 | 1.57E-04 | 5.6692266 | up | LOC157562 | BX649145 | hypothetical protein LOC157562 |
| A_24_P941487 | 2.44E-05 | 5.6541104 | up | ZNF761 | NM_001008401 | zinc finger protein 761 |
| A_19_P00326917 | 1.03E-05 | 5.6236424 | up |  |  |  |
| A_19_P00321865 | 2.81E-06 | 5.489123 | up |  |  |  |
| A_23_P65797 | 4.18E-05 | 5.418629 | up | KLHL25 | NM_022480 | kelch-like 25 (Drosophila) |
| A_32_P123514 | 1.58E-04 | 5.4128027 | up | PABPC4L | NM_001114734 | poly(A) binding protein. cytoplasmic 4-like |
| A_33_P3214948 | 2.49E-05 | 5.1789107 | up | SPOCK2 | NM_014767 | sparc/osteonectin. cwcv and kazal-like domains proteoglycan (testican) 2 |
| A_23_P159775 | 1.41E-04 | 5.1115174 | up | GABRE | NM_004961 | gamma-aminobutyric acid (GABA) A receptor. epsilon |
| A_24_P102981 | 8.83E-07 | 5.0652623 | up | DNAJB2 | NM_006736 | DnaJ (Hsp40) homolog. subfamily B. member 2 |
| A_24_P166311 | 2.37E-05 | 5.0124197 | up | C5orf54 | NM_022090 | chromosome 5 open reading frame 54 |
| A_33_P3236778 | 1.34E-05 | 4.97363 | up |  |  |  |
| A_33_P3385436 | 3.56E-05 | 4.89028 | up | PLAC8L1 | NM_001029869 | PLAC8-like 1 |
| A_33_P3417452 | 3.37E-06 | 4.889631 | up | ZGLP1 | NM_001103167 | zinc finger. GATA-like protein 1 |
| A_33_P3269203 | 1.01E-04 | 4.657137 | up | SERPINH1 | NM_001235 | serpin peptidase inhibitor. clade H (heat shock protein 47). member 1. (collagen binding protein 1) |
| A_19_P00321751 | 1.98E-04 | 4.627576 | up |  |  |  |
| A_23_P130764 | 1.16E-05 | 4.5825753 | up | KCNJ14 | NM_170720 | potassium inwardly-rectifying channel. subfamily J. member 14 |
| A_33_P3849600 | 1.15E-05 | 4.4985313 | up | HSP90AA2 | M30627 | heat shock protein 90kDa alpha (cytosolic). class A member 2 |
| A_33_P3289422 | 1.27E-05 | 4.4914794 | up | ZNF765 | NM_001040185 | zinc finger protein 765 |
| A_23_P311616 | 3.78E-05 | 4.4786716 | up | JMJD6 | NM_015167 | jumonji domain containing 6 |
| A_33_P3222753 | 6.80E-05 | 4.425918 | up | ZNF137 | NR_023311 | zinc finger protein 137 |
| A_19_P00328670 | 5.06E-05 | 4.408048 | up |  |  |  |
| A_23_P22263 | 2.35E-05 | 4.384706 | up | BANP | NM_079837 | BTG3 associated nuclear protein |
| A_32_P199252 | 1.32E-05 | 4.3813567 | up | HSP90AA1 | NM_001017963 | heat shock protein 90kDa alpha (cytosolic). class A member 1 |
| A_32_P41065 | 2.64E-05 | 4.3676867 | up | TMCC1 | NM_001017395 | transmembrane and coiled-coil domain family 1 |
| A_23_P256641 | 3.65E-04 | 4.3300915 | up | KCNE1L | NM_012282 | KCNE1-like |
| A_33_P3273309 | 4.09E-05 | 4.307111 | up | ZNF765 | NM_001040185 | zinc finger protein 765 |
| A_23_P432034 | 1.79E-06 | 4.3068867 | up | CCDC117 | NM_173510 | coiled-coil domain containing 117 |
| A_23_P44724 | 1.54E-05 | 4.2986846 | up | CSRP2 | NM_001321 | cysteine and glycine-rich protein 2 |
| A_33_P3739260 | 6.93E-05 | 4.238484 | up | CAP2 | NM_006366 | CAP. adenylate cyclase-associated protein. 2 (yeast) |
| A_33_P3341424 | 1.16E-06 | 4.2270756 | up | MYO10 | NM_012334 | myosin X |
| A_23_P363936 | 3.94E-05 | 4.1983404 | up | HSPA4L | NM_014278 | heat shock 70kDa protein 4-like |
| A_24_P45379 | 6.96E-05 | 4.139569 | up | CACYBP | NM_014412 | calcyclin binding protein |
| A_23_P162874 | 5.25E-06 | 4.1126337 | up | HSP90AA1 | NM_005348 | heat shock protein 90kDa alpha (cytosolic). class A member 1 |
| A_23_P24275 | 8.77E-05 | 4.0915046 | up | C10orf110 | NR_027709 | chromosome 10 open reading frame 110 |
| A_23_P207037 | 4.65E-06 | 4.09133 | up | CD300A | NM_007261 | CD300a molecule |
| A_19_P00323873 | 2.04E-04 | 4.062632 | up |  |  |  |
| A_32_P514599 | 1.47E-05 | 4.0604906 | up |  |  |  |
| A_23_P209519 | 3.57E-05 | 4.0470543 | up | DNAJB2 | NM_001039550 | DnaJ (Hsp40) homolog. subfamily B. member 2 |
| A_33_P3399380 | 2.70E-05 | 4.0306253 | up |  |  |  |
| A_33_P3352767 | 1.02E-06 | 3.974175 | up | MC1R | NM_002386 | melanocortin 1 receptor (alpha melanocyte stimulating hormone receptor) |
| A_24_P307384 | 1.11E-06 | 3.9658723 | up |  |  |  |
| A_32_P79115 | 3.33E-05 | 3.9656508 | up | LOC100133075 | XR_039086 | hypothetical LOC100133075 |
| A_19_P00325050 | 1.50E-07 | 3.931666 | up |  |  |  |
| A_32_P71788 | 7.05E-07 | 3.9186695 | up | FKBP4 | NM_002014 | FK506 binding protein 4. 59kDa |
| A_19_P00317360 | 1.11E-05 | 3.9175858 | up |  |  |  |
| A_23_P35564 | 1.05E-04 | 3.9152157 | up | SEC31B | NM_015490 | SEC31 homolog B (S. cerevisiae) |
| A_24_P56317 | 3.11E-06 | 3.90081 | up | MBNL2 | NM_144778 | muscleblind-like 2 (Drosophila) |
| A_23_P259621 | 1.87E-04 | 3.8973079 | up | LAT2 | NM_032464 | linker for activation of T cells family. member 2 |
| A_33_P3211513 | 2.63E-04 | 3.8855371 | up | CLK1 | NM_001162407 | CDC-like kinase 1 |
| A_33_P3309924 | 1.34E-04 | 3.8853648 | up | HDAC2 | NM_001527 | histone deacetylase 2 |
| A_32_P64200 | 1.05E-04 | 3.8659554 | up | GUCA1B | NM_002098 | guanylate cyclase activator 1B (retina) |
| A_33_P3391076 | 3.70E-04 | 3.849854 | up | MBNL2 | NM_207304 | muscleblind-like 2 (Drosophila) |
| A_23_P329261 | 2.94E-04 | 3.8401706 | up | KCNJ2 | NM_000891 | potassium inwardly-rectifying channel. subfamily J. member 2 |
| A_23_P365189 | 7.95E-07 | 3.7638884 | up | CAMTA2 | NM_015099 | calmodulin binding transcription activator 2 |
| A_33_P3263432 | 1.23E-04 | 3.7618775 | up | ITGA10 | NM_003637 | integrin. alpha 10 |
| A_24_P303647 | 2.93E-05 | 3.7474012 | up | C7orf60 | NM_152556 | chromosome 7 open reading frame 60 |
| A_33_P3328637 | 1.96E-04 | 3.7229712 | up |  |  |  |
| A_33_P3601163 | 1.03E-04 | 3.7154565 | up | LOC147727 | NR_024333 | hypothetical LOC147727 |
| A_23_P129466 | 2.41E-05 | 3.7047653 | up | ATF7IP2 | NM_024997 | activating transcription factor 7 interacting protein 2 |
| A_23_P334870 | 1.75E-04 | 3.678116 | up | TMEM217 | NM_145316 | transmembrane protein 217 |
| A_33_P3215288 | 6.45E-06 | 3.676358 | up | LOC284757 | AK128288 | hypothetical protein LOC284757 |
| A_23_P364465 | 2.33E-04 | 3.6610258 | up | GPBP1 | NM_022913 | GC-rich promoter binding protein 1 |
| A_19_P00802221 | 1.01E-04 | 3.65602 | up |  |  |  |
| A_33_P3404316 | 3.99E-04 | 3.6396737 | up | LOC399959 | NR_024430 | hypothetical LOC399959 |
| A_33_P3353030 | 9.17E-05 | 3.6278293 | up | UCN | NM_003353 | urocortin |
| A_23_P350005 | 5.75E-05 | 3.6078448 | up | TRIML2 | NM_173553 | tripartite motif family-like 2 |
| A_32_P212058 | 5.10E-06 | 3.579287 | up | C3orf34 | NM_032898 | chromosome 3 open reading frame 34 |
| A_19_P00809154 | 5.42E-06 | 3.5742536 | up |  |  |  |
| A_24_P84428 | 8.46E-06 | 3.5515318 | up | CACYBP | NM_014412 | calcyclin binding protein |
| A_23_P426663 | 5.54E-04 | 3.5129852 | up | MITF | NM_198159 | microphthalmia-associated transcription factor |
| A_23_P375281 | 0.00101131 | 3.5114894 | up | TRPV1 | NM_080706 | transient receptor potential cation channel. subfamily V. member 1 |
| A_33_P3299882 | 1.66E-04 | 3.5008059 | up | UQCRB | NM_006294 | ubiquinol-cytochrome c reductase binding protein |
| A_23_P27381 | 3.72E-05 | 3.4817653 | up | TSHZ1 | NM_005786 | teashirt zinc finger homeobox 1 |
| A_33_P3219601 | 3.00E-06 | 3.4487846 | up | ABL2 | NM_001100108 | v-abl Abelson murine leukemia viral oncogene homolog 2 (arg. Abelson-related gene) |
| A_19_P00319448 | 2.02E-06 | 3.4118721 | up |  |  |  |
| A_32_P114574 | 1.27E-05 | 3.405849 | up | CACYBP | NM_014412 | calcyclin binding protein |
| A_23_P361448 | 6.00E-06 | 3.3865001 | up | SESN3 | NM_144665 | sestrin 3 |
| A_19_P00319456 | 5.65E-06 | 3.3848448 | up |  |  |  |
| A_24_P876522 | 8.38E-05 | 3.3474832 | up | GPX8 | NM_001008397 | glutathione peroxidase 8 (putative) |
| A_33_P3413855 | 1.35E-05 | 3.3438687 | up | C7orf60 | NM_152556 | chromosome 7 open reading frame 60 |
| A_23_P154022 | 7.65E-05 | 3.3175857 | up | C2orf44 | NM_025203 | chromosome 2 open reading frame 44 |
| A_33_P3323928 | 1.72E-06 | 3.3077893 | up | C10orf75 | NR_026762 | chromosome 10 open reading frame 75 |
| A_33_P3338733 | 4.90E-04 | 3.295123 | up | MITF | NM_198159 | microphthalmia-associated transcription factor |
| A_23_P64860 | 1.43E-05 | 3.2799666 | up | SELPLG | NM_003006 | selectin P ligand |
| A_33_P3345031 | 1.26E-04 | 3.2759314 | up | LOC645978 | XR_017572 | similar to alkaline ceramidase 2 |
| A_19_P00321110 | 5.71E-05 | 3.2361567 | up |  |  |  |
| A_33_P3262020 | 4.55E-05 | 3.2261162 | up | C8G | NM_000606 | complement component 8. gamma polypeptide |
| A_33_P3280009 | 1.68E-04 | 3.219283 | up |  |  |  |
| A_23_P356616 | 7.87E-04 | 3.2123153 | up | ABTB2 | NM_145804 | ankyrin repeat and BTB (POZ) domain containing 2 |
| A_23_P61674 | 2.57E-05 | 3.207084 | up | CLK4 | NM_020666 | CDC-like kinase 4 |
| A_33_P3304668 | 0.00120266 | 3.1996348 | up | COL1A1 | NM_000088 | collagen. type I. alpha 1 |
| A_33_P3300273 | 7.65E-05 | 3.1943436 | up | C1orf52 | NM_198077 | chromosome 1 open reading frame 52 |
| A_23_P142708 | 3.19E-04 | 3.1893778 | up | LMAN2L | NM_030805 | lectin. mannose-binding 2-like |
| A_33_P3395971 | 2.12E-04 | 3.1800163 | up | CTU1 | NM_145232 | cytosolic thiouridylase subunit 1 homolog (S. pombe) |
| A_33_P3351559 | 1.92E-05 | 3.1773596 | up | ETNK1 | NM_018638 | ethanolamine kinase 1 |
| A_23_P128744 | 2.21E-04 | 3.1281044 | up | BDKRB1 | NM_000710 | bradykinin receptor B1 |
| A_23_P112187 | 9.19E-05 | 3.1220076 | up | FIBCD1 | NM_032843 | fibrinogen C domain containing 1 |
| A_19_P00332340 | 1.72E-05 | 3.101769 | up |  |  |  |
| A_23_P110643 | 4.45E-07 | 3.0959964 | up | CDKL3 | NM_016508 | cyclin-dependent kinase-like 3 |
| A_19_P00322132 | 4.81E-07 | 3.0778286 | up |  |  |  |
| A_23_P137381 | 1.18E-04 | 3.0558288 | up | ID3 | NM_002167 | inhibitor of DNA binding 3. dominant negative helix-loop-helix protein |
| A_33_P3390758 | 1.16E-06 | 3.0480838 | up | HSPA8 | NM_153201 | heat shock 70kDa protein 8 |
| A_19_P00800237 | 3.94E-04 | 3.0330725 | up |  |  |  |
| A_23_P431268 | 8.36E-04 | 3.0204825 | up | PLEKHA6 | NM_014935 | pleckstrin homology domain containing. family A member 6 |
| A_24_P237586 | 1.37E-04 | 3.011613 | up | ANKRD37 | NM_181726 | ankyrin repeat domain 37 |
| A_33_P3379916 | 8.54E-04 | 2.994473 | up | GLS | NM_014905 | glutaminase |
| A_24_P55496 | 2.85E-04 | 2.9943423 | up | OSR2 | NM_053001 | odd-skipped related 2 (Drosophila) |
| A_24_P178631 | 4.10E-05 | 2.992685 | up |  | AK027667 |  |
| A_32_P25273 | 3.76E-06 | 2.9841664 | up | HSPD1 | NM_002156 | heat shock 60kDa protein 1 (chaperonin) |
| A_23_P8339 | 2.43E-06 | 2.9818919 | up | MRPL18 | NM_014161 | mitochondrial ribosomal protein L18 |
| A_33_P3619171 | 7.22E-04 | 2.9807804 | up | PMAIP1 | NM_021127 | phorbol-12-myristate-13-acetate-induced protein 1 |
| A_23_P385063 | 9.10E-05 | 2.9734235 | up | DNAJB6 | NM_058246 | DnaJ (Hsp40) homolog. subfamily B. member 6 |
| A_19_P00324814 | 5.54E-04 | 2.9706175 | up |  |  |  |
| A_32_P61684 | 0.00229914 | 2.9660795 | up | PAG1 | NM_018440 | phosphoprotein associated with glycosphingolipid microdomains 1 |
| A_24_P153456 | 0.00286583 | 2.962745 | up | ZDHHC11 | NM_024786 | zinc finger. DHHC-type containing 11 |
| A_33_P3282840 | 2.72E-05 | 2.9560504 | up | RPS29 | NM_001030001 | ribosomal protein S29 |
| A_32_P135336 | 0.00159781 | 2.93938 | up | LOC388242 | NR_002556 | coiled-coil domain containing 101 pseudogene |
| A_33_P3296497 | 4.73E-04 | 2.8856902 | up |  | BC063596 |  |
| A_23_P71727 | 3.17E-06 | 2.8825352 | up | CKS2 | NM_001827 | CDC28 protein kinase regulatory subunit 2 |
| A_23_P117599 | 2.66E-06 | 2.8772464 | up | AHSA1 | NM_012111 | AHA1. activator of heat shock 90kDa protein ATPase homolog 1 (yeast) |
| A_33_P3407985 | 1.30E-05 | 2.8548174 | up | TMEM99 | NM_145274 | transmembrane protein 99 |
| A_23_P320225 | 4.75E-05 | 2.826504 | up | EVC2 | NM_147127 | Ellis van Creveld syndrome 2 |
| A_19_P00322690 | 4.29E-04 | 2.8186293 | up |  |  |  |
| A_33_P3274397 | 3.40E-05 | 2.8148177 | up | CHM | NM_001145414 | choroideremia (Rab escort protein 1) |
| A_23_P106391 | 7.19E-05 | 2.809145 | up | THAP10 | NM_020147 | THAP domain containing 10 |
| A_23_P144337 | 2.03E-04 | 2.792439 | up | CCRN4L | NM_012118 | CCR4 carbon catabolite repression 4-like (S. cerevisiae) |
| A_33_P3353242 | 1.68E-04 | 2.7806816 | up | HSPB1 | NM_001540 | heat shock 27kDa protein 1 |
| A_19_P00323375 | 6.21E-05 | 2.7783225 | up |  |  |  |
| A_33_P3330404 | 1.51E-04 | 2.765178 | up | FAM180B | NM_001164379 | family with sequence similarity 180. member B |
| A_24_P782308 | 0.00217325 | 2.7494533 | up | NEDD4L | NM_001144967 | neural precursor cell expressed. developmentally down-regulated 4-like |
| A_23_P27215 | 5.65E-04 | 2.7424445 | up | UBB | NM_018955 | ubiquitin B |
| A_24_P583040 | 7.71E-05 | 2.7233615 | up | C17orf67 | XM_001718395 | chromosome 17 open reading frame 67 |
| A_24_P940115 | 0.00385828 | 2.7069464 | up | DLC1 | NM_182643 | deleted in liver cancer 1 |
| A_33_P3340154 | 2.12E-04 | 2.701536 | up | DGCR14 | NM_022719 | DiGeorge syndrome critical region gene 14 |
| A_24_P152188 | 2.20E-04 | 2.6885436 | up | PRICKLE2 | NM_198859 | prickle homolog 2 (Drosophila) |
| A_33_P3354796 | 2.38E-04 | 2.6885312 | up | FLJ42627 | NR_024492 | hypothetical LOC645644 |
| A_23_P43157 | 6.80E-04 | 2.6795297 | up | MYBL1 | NM_001080416 | v-myb myeloblastosis viral oncogene homolog (avian)-like 1 |
| A_24_P79070 | 3.97E-05 | 2.676245 | up | GNG8 | NM_033258 | guanine nucleotide binding protein (G protein). gamma 8 |
| A_33_P3299319 | 2.05E-04 | 2.675945 | up | RPL28 | NM_001136137 | ribosomal protein L28 |
| A_24_P778836 | 6.63E-05 | 2.6657512 | up | RSL1D1 | NM_015659 | ribosomal L1 domain containing 1 |
| A_23_P6596 | 1.63E-04 | 2.6555274 | up | HES1 | NM_005524 | hairy and enhancer of split 1. (Drosophila) |
| A_23_P372467 | 6.09E-04 | 2.6473424 | up | AHSA2 | NM_152392 | AHA1. activator of heat shock 90kDa protein ATPase homolog 2 (yeast) |
| A_23_P166280 | 4.33E-05 | 2.6455266 | up | ICOSLG |  | inducible T-cell co-stimulator ligand |
| A_33_P3210139 | 4.82E-06 | 2.6417496 | up | PCF11 | NM_015885 | PCF11. cleavage and polyadenylation factor subunit. homolog (S. cerevisiae) |
| A_24_P108311 | 8.73E-04 | 2.6357005 | up | NEDD4L | NM_015277 | neural precursor cell expressed. developmentally down-regulated 4-like |
| A_24_P110558 | 4.26E-04 | 2.626492 | up | C5orf53 | NM_001007189 | chromosome 5 open reading frame 53 |
| A_33_P3362143 | 3.67E-04 | 2.6044557 | up | LOC284900 | NR_026962 | hypothetical LOC284900 |
| A_32_P139894 | 0.00133531 | 2.5960817 | up | ABL2 | NM_001100108 | v-abl Abelson murine leukemia viral oncogene homolog 2 (arg. Abelson-related gene) |
| A_32_P150735 | 4.26E-04 | 2.595806 | up |  |  |  |
| A_23_P128372 | 4.21E-06 | 2.5922213 | up | FKBP4 | NM_002014 | FK506 binding protein 4. 59kDa |
| A_33_P3319880 | 5.75E-04 | 2.5885053 | up | EVC2 | NM_001166136 | Ellis van Creveld syndrome 2 |
| A_33_P3232011 | 0.00380446 | 2.5815 | up | RAB17 | NM_022449 | RAB17. member RAS oncogene family |
| A_23_P374782 | 0.00127725 | 2.5813732 | up | SH3KBP1 | NM_001024666 | SH3-domain kinase binding protein 1 |
| A_33_P3807593 | 4.61E-04 | 2.5795634 | up | LOC286437 | AK093652 | hypothetical protein LOC286437 |
| A_19_P00810012 | 7.96E-04 | 2.5784192 | up |  |  |  |
| A_24_P919452 | 7.81E-04 | 2.5652702 | up | LOC729839 | XR_016021 | similar to DTW domain containing 2 |
| A_33_P3237729 | 0.00243709 | 2.5596716 | up | PDLIM4 | NM_003687 | PDZ and LIM domain 4 |
| A_23_P139143 | 1.67E-05 | 2.55801 | up | STX3 | NM_004177 | syntaxin 3 |
| A_23_P319617 | 2.81E-04 | 2.5496929 | up | CHST7 | NM_019886 | carbohydrate (N-acetylglucosamine 6-O) sulfotransferase 7 |
| A_32_P122940 | 0.00104401 | 2.546974 | up | LOC642852 | NR_026943 | hypothetical LOC642852 |
| A_33_P3395738 | 2.50E-04 | 2.5453064 | up | LOC100131601 | AK127004 | similar to hCG1980470 |
| A_33_P3665777 | 5.95E-07 | 2.542213 | up | HSP90AA1 | NM_001017963 | heat shock protein 90kDa alpha (cytosolic). class A member 1 |
| A_24_P396327 | 3.65E-06 | 2.5418546 | up | TYW3 | NM_138467 | tRNA-yW synthesizing protein 3 homolog (S. cerevisiae) |
| A_24_P1255 | 1.90E-04 | 2.5404902 | up | BCCIP | NM_078469 | BRCA2 and CDKN1A interacting protein |
| A_19_P00318719 | 3.46E-04 | 2.5363102 | up |  |  |  |
| A_33_P3329834 | 7.24E-06 | 2.5283034 | up | PRPF38B | NM_018061 | PRP38 pre-mRNA processing factor 38 (yeast) domain containing B |
| A_33_P3718352 | 2.47E-04 | 2.5247438 | up | LOC170425 | AK056561 | hypothetical protein LOC170425 |
| A_23_P313223 | 7.36E-05 | 2.524577 | up | C11orf84 | NM_138471 | chromosome 11 open reading frame 84 |
| A_24_P50753 | 3.22E-05 | 2.521049 | up | NUDT4 | NM_199040 | nudix (nucleoside diphosphate linked moiety X)-type motif 4 |
| A_33_P3540143 | 8.26E-04 | 2.520553 | up | IL17RA | AK056836 | interleukin 17 receptor A |
| A_23_P356109 | 7.59E-06 | 2.5123346 | up | ATXN7L1 | NM_152749 | ataxin 7-like 1 |
| A_24_P167338 | 2.86E-04 | 2.5041478 | up | RAB30 | NM_014488 | RAB30. member RAS oncogene family |
| A_32_P92783 | 1.12E-05 | 2.5020888 | up | STIP1 | NM_006819 | stress-induced-phosphoprotein 1 |
| A_23_P339053 | 6.11E-06 | 2.5008783 | up |  |  |  |
| A_24_P327815 | 1.40E-05 | 2.500574 | up | STIP1 | NM_006819 | stress-induced-phosphoprotein 1 |
| A_23_P13772 | 2.03E-04 | 2.497417 | up | TBX3 | NM_016569 | T-box 3 |
| A_23_P79441 | 8.42E-06 | 2.49516 | up | C2orf42 | NM_017880 | chromosome 2 open reading frame 42 |
| A_32_P41026 | 0.00116765 | 2.4838924 | up | SC5DL | NM_001024956 | sterol-C5-desaturase (ERG3 delta-5-desaturase homolog. S. cerevisiae)-like |
| A_33_P3287646 | 6.96E-04 | 2.4837744 | up | HSPB1 | NM_001540 | heat shock 27kDa protein 1 |
| A_33_P3216232 | 0.00233345 | 2.481016 | up | ITGB1BP1 | NM_004763 | integrin beta 1 binding protein 1 |
| A_33_P3589543 | 2.24E-05 | 2.4686687 | up |  | AK093839 |  |
| A_23_P427217 | 0.0015662 | 2.4637897 | up | JMJD1C | NM_032776 | jumonji domain containing 1C |
| A_23_P213319 | 0.00405299 | 2.4583046 | up | ADAMTS6 | NM_197941 | ADAM metallopeptidase with thrombospondin type 1 motif. 6 |
| A_24_P252497 | 0.0052625 | 2.4551167 | up | TRIB1 | NM_025195 | tribbles homolog 1 (Drosophila) |
| A_33_P3380682 | 8.12E-05 | 2.4463031 | up | TBPL1 | NM_004865 | TBP-like 1 |
| A_33_P3242483 | 3.48E-04 | 2.4427578 | up | ARHGAP5 | NM_001030055 | Rho GTPase activating protein 5 |
| A_23_P305759 | 4.06E-04 | 2.4398596 | up | ABHD3 | NM_138340 | abhydrolase domain containing 3 |
| A_23_P339818 | 0.00125168 | 2.438787 | up | ARRDC4 | NM_183376 | arrestin domain containing 4 |
| A_23_P168276 | 4.02E-05 | 2.4375203 | up | TBPL1 | NM_004865 | TBP-like 1 |
| A_19_P00806882 | 1.84E-05 | 2.4306438 | up |  |  |  |
| A_19_P00811181 | 9.55E-06 | 2.4273496 | up |  |  |  |
| A_33_P3352887 | 1.39E-04 | 2.4249344 | up | LOC388692 | NR_027002 | hypothetical LOC388692 |
| A_24_P239606 | 0.00226548 | 2.4101083 | up | GADD45B | NM_015675 | growth arrest and DNA-damage-inducible. beta |
| A_32_P355396 | 8.29E-05 | 2.4027681 | up | TECPR2 | NM_014844 | tectonin beta-propeller repeat containing 2 |
| A_24_P67946 | 2.76E-04 | 2.3946593 | up | NUDT4 | NM_199040 | nudix (nucleoside diphosphate linked moiety X)-type motif 4 |
| A_32_P128656 | 2.88E-05 | 2.393808 | up | MID1 | NM_000381 | midline 1 (Opitz/BBB syndrome) |
| A_33_P3273272 | 9.33E-05 | 2.3923705 | up | ZNF701 | NM_018260 | zinc finger protein 701 |
| A_33_P3356517 | 0.00110391 | 2.3908465 | up |  |  |  |
| A_24_P248606 | 4.94E-04 | 2.389747 | up | ACSL3 | NM_004457 | acyl-CoA synthetase long-chain family member 3 |
| A_23_P48295 | 2.30E-05 | 2.3874602 | up | CDADC1 | NM_030911 | cytidine and dCMP deaminase domain containing 1 |
| A_33_P3240229 | 1.79E-05 | 2.386294 | up | CREBBP | NM_004380 | CREB binding protein |
| A_32_P121085 | 3.24E-04 | 2.3854227 | up | DOK3 | NM_001144875 | docking protein 3 |
| A_33_P3305093 | 6.39E-05 | 2.3694718 | up | ZGLP1 | NM_001103167 | zinc finger. GATA-like protein 1 |
| A_33_P3374463 | 1.30E-05 | 2.3660812 | up | MFAP3 | NM_005927 | microfibrillar-associated protein 3 |
| A_23_P88691 | 1.17E-05 | 2.3579364 | up | CHRNA5 | NM_000745 | cholinergic receptor. nicotinic. alpha 5 |
| A_33_P3216433 | 8.98E-05 | 2.3454013 | up | LOC147804 | NR_003148 | tropomyosin 3 pseudogene |
| A_19_P00806974 | 1.13E-04 | 2.3410478 | up |  |  |  |
| A_24_P136470 | 0.00104216 | 2.3397827 | up | BTN2A3 | NR_027795 | butyrophilin. subfamily 2. member A3 |
| A_33_P3325306 | 4.98E-05 | 2.3355365 | up |  |  |  |
| A_33_P3349947 | 6.80E-04 | 2.331791 | up | TCP1 | NM_030752 | t-complex 1 |
| A_23_P218817 | 0.00445768 | 2.324564 | up | CPT1B | NM_152246 | carnitine palmitoyltransferase 1B (muscle) |
| A_23_P68211 | 5.69E-06 | 2.3163867 | up | SPR | NM_003124 | sepiapterin reductase (7.8-dihydrobiopterin:NADP+ oxidoreductase) |
| A_24_P185854 | 5.76E-04 | 2.3156388 | up | DMD | NM_004010 | dystrophin |
| A_23_P47058 | 6.01E-05 | 2.3138323 | up | CUZD1 | NM_022034 | CUB and zona pellucida-like domains 1 |
| A_33_P3294901 | 0.00339121 | 2.313595 | up | SULT1A4 | NM_001017389 | sulfotransferase family. cytosolic. 1A. phenol-preferring. member 4 |
| A_23_P308954 | 3.72E-05 | 2.3112783 | up | BHLHB9 | NM_030639 | basic helix-loop-helix domain containing. class B. 9 |
| A_32_P137266 | 2.51E-04 | 2.307673 | up | EFCAB7 | NM_032437 | EF-hand calcium binding domain 7 |
| A_33_P3408054 | 6.54E-04 | 2.3070614 | up | HSP90AB2P | NR_003132 | heat shock protein 90kDa alpha (cytosolic). class B member 2 (pseudogene) |
| A_23_P424597 | 1.26E-04 | 2.3029537 | up | C19orf25 | NM_152482 | chromosome 19 open reading frame 25 |
| A_23_P214587 | 3.88E-04 | 2.2997572 | up | TRIM26 | NM_003449 | tripartite motif-containing 26 |
| A_23_P256158 | 6.30E-04 | 2.2971928 | up | ADRA2C | NM_000683 | adrenergic. alpha-2C-. receptor |
| A_19_P00808377 | 2.83E-05 | 2.2969062 | up |  |  |  |
| A_33_P3278043 | 3.86E-04 | 2.295654 | up | RG9MTD2 | NM_152292 | RNA (guanine-9-) methyltransferase domain containing 2 |
| A_24_P182494 | 0.00342831 | 2.293456 | up | DUSP10 | NM_007207 | dual specificity phosphatase 10 |
| A_32_P168442 | 0.00456396 | 2.2860613 | up | HOTAIR | NR_003716 | hox transcript antisense RNA (non-protein coding) |
| A_23_P435697 | 1.67E-05 | 2.2860396 | up | C10orf137 | NM_015608 | chromosome 10 open reading frame 137 |
| A_24_P379750 | 0.00291973 | 2.2854717 | up | MXD1 | NM_002357 | MAX dimerization protein 1 |
| A_24_P169688 | 1.71E-05 | 2.283755 | up | MICB | NM_005931 | MHC class I polypeptide-related sequence B |
| A_32_P210202 | 7.42E-05 | 2.283201 | up | E2F7 | NM_203394 | E2F transcription factor 7 |
| A_33_P3299314 | 0.00204857 | 2.2826753 | up | RPL28 | NM_001136134 | ribosomal protein L28 |
| A_23_P128084 | 1.38E-04 | 2.2797706 | up | ITGA7 | NM_002206 | integrin. alpha 7 |
| A_24_P350744 | 2.82E-05 | 2.2757385 | up | KIAA0907 | NM_014949 | KIAA0907 |
| A_23_P100420 | 1.48E-06 | 2.263809 | up | ZCCHC14 | NM_015144 | zinc finger. CCHC domain containing 14 |
| A_23_P56922 | 3.78E-05 | 2.262034 | up | HSPE1 | NM_002157 | heat shock 10kDa protein 1 (chaperonin 10) |
| A_33_P3216994 | 3.80E-04 | 2.260273 | up | HERC4 | NM_015601 | hect domain and RLD 4 |
| A_24_P56240 | 8.91E-05 | 2.244059 | up | CPNE8 | NM_153634 | copine VIII |
| A_33_P3247624 | 2.17E-04 | 2.2404757 | up | REP15 | NM_001029874 | RAB15 effector protein |
| A_33_P3261505 | 1.87E-04 | 2.2373078 | up | KIAA1632 | NM_020964 | KIAA1632 |
| A_33_P3218625 | 3.80E-04 | 2.2359064 | up | C9orf7 | NM_017586 | chromosome 9 open reading frame 7 |
| A_24_P372553 | 2.67E-06 | 2.2352192 | up | C10orf137 | NM_015608 | chromosome 10 open reading frame 137 |
| A_33_P3411145 | 3.52E-05 | 2.2260036 | up | LOC285550 | NM_001145191 | hypothetical protein LOC285550 |
| A_33_P3260500 | 8.54E-04 | 2.2256594 | up | BREA2 | NR_015445 | breast cancer estrogen-induced apoptosis 2 |
| A_24_P497186 | 0.00106565 | 2.2144902 | up | IRF2BP2 | NM_182972 | interferon regulatory factor 2 binding protein 2 |
| A_33_P3762918 | 6.60E-06 | 2.2111807 | up | LOC100216546 | AK123595 | hypothetical LOC100216546 |
| A_23_P79962 | 1.60E-04 | 2.207001 | up | MKKS | NM_170784 | McKusick-Kaufman syndrome |
| A_23_P58009 | 7.64E-05 | 2.2064328 | up | C3orf52 | NM_024616 | chromosome 3 open reading frame 52 |
| A_33_P3259615 | 1.16E-04 | 2.203831 | up | TTC36 | NM_001080441 | tetratricopeptide repeat domain 36 |
| A_19_P00325912 | 0.00258935 | 2.2006717 | up |  |  |  |
| A_19_P00327355 | 0.0045522 | 2.1984446 | up |  |  |  |
| A_19_P00318213 | 4.57E-04 | 2.1945517 | up |  |  |  |
| A_33_P3221999 | 0.00596516 | 2.1893 | up | GSDMB | NM_001165958 | gasdermin B |
| A_23_P39766 | 9.37E-04 | 2.188801 | up | GLS | NM_014905 | glutaminase |
| A_33_P3257503 | 1.89E-06 | 2.1854486 | up | LOC387647 | NR_003930 | patched domain containing 3 pseudogene |
| A_23_P201567 | 8.81E-07 | 2.184417 | up | PRPF38B | NM_018061 | PRP38 pre-mRNA processing factor 38 (yeast) domain containing B |
| A_23_P64689 | 2.44E-05 | 2.1835911 | up | PAN2 | NM_014871 | PAN2 poly(A) specific ribonuclease subunit homolog (S. cerevisiae) |
| A_23_P204630 | 0.00878755 | 2.1822784 | up | NTN4 | NM_021229 | netrin 4 |
| A_23_P30363 | 3.25E-04 | 2.1775177 | up | P4HA2 | NM_004199 | prolyl 4-hydroxylase. alpha polypeptide II |
| A_24_P286114 | 3.81E-04 | 2.17609 | up | SLC1A3 | NM_004172 | solute carrier family 1 (glial high affinity glutamate transporter). member 3 |
| A_23_P251785 | 2.73E-04 | 2.1757588 | up | NARG1L | NM_024561 | NMDA receptor regulated 1-like |
| A_23_P135857 | 0.00572155 | 2.171924 | up | EIF2AK3 | NM_004836 | eukaryotic translation initiation factor 2-alpha kinase 3 |
| A_19_P00809624 | 5.15E-05 | 2.170742 | up |  |  |  |
| A_33_P3214481 | 8.99E-04 | 2.1678905 | up | P4HA1 | NM_001142595 | prolyl 4-hydroxylase. alpha polypeptide I |
| A_32_P9468 | 0.00302956 | 2.1669693 | up | COX19 | NM_001031617 | COX19 cytochrome c oxidase assembly homolog (S. cerevisiae) |
| A_33_P3281010 | 6.43E-04 | 2.166023 | up | FAM95B1 | AK125850 | family with sequence similarity 95. member B1 |
| A_23_P57856 | 0.00145528 | 2.1660213 | up | BCL6 | NM_001130845 | B-cell CLL/lymphoma 6 |
| A_23_P97700 | 0.00210749 | 2.1658719 | up | TXNIP | NM_006472 | thioredoxin interacting protein |
| A_33_P3305974 | 6.55E-05 | 2.165561 | up | SNAP23 | NM_003825 | synaptosomal-associated protein. 23kDa |
| A_33_P3304107 | 3.35E-04 | 2.1633558 | up | KLHL28 | NM_017658 | kelch-like 28 (Drosophila) |
| A_19_P00801511 | 3.02E-04 | 2.162683 | up |  |  |  |
| A_24_P333421 | 0.00568893 | 2.1599886 | up | ZNF862 | NM_001099220 | zinc finger protein 862 |
| A_23_P359647 | 0.00188285 | 2.1597319 | up | NFAT5 | NM_138714 | nuclear factor of activated T-cells 5. tonicity-responsive |
| A_19_P00327929 | 9.07E-05 | 2.156836 | up |  |  |  |
| A_23_P10291 | 5.42E-05 | 2.1553965 | up | CTSE | NM_001910 | cathepsin E |
| A_32_P100258 | 0.00557985 | 2.1470594 | up | hCG_2008140 | NR_024279 | hypothetical LOC729614 |
| A_24_P137522 | 0.00238993 | 2.1440792 | up | USP53 | NM_019050 | ubiquitin specific peptidase 53 |
| A_24_P114255 | 0.00238747 | 2.1396775 | up | MBOAT2 | NM_138799 | membrane bound O-acyltransferase domain containing 2 |
| A_24_P823684 | 2.91E-05 | 2.136701 | up | HSP90AB1 | NM_007355 | heat shock protein 90kDa alpha (cytosolic). class B member 1 |
| A_33_P3324890 | 6.61E-05 | 2.1341915 | up | HSP90AB2P | NR_003132 | heat shock protein 90kDa alpha (cytosolic). class B member 2 (pseudogene) |
| A_23_P318296 | 1.50E-04 | 2.1291807 | up | PLEKHA8 | NM_032639 | pleckstrin homology domain containing. family A (phosphoinositide binding specific) member 8 |
| A_32_P114896 | 2.05E-04 | 2.1255698 | up | PTGES3 | NM_006601 | prostaglandin E synthase 3 (cytosolic) |
| A_23_P119916 | 4.58E-04 | 2.123948 | up | WNT6 | NM_006522 | wingless-type MMTV integration site family. member 6 |
| A_33_P3286953 | 3.13E-04 | 2.1215327 | up | ADAMTS6 | NM_197941 | ADAM metallopeptidase with thrombospondin type 1 motif. 6 |
| A_33_P3882659 | 2.46E-05 | 2.1203215 | up | HSP90AB5P | AY956766 | heat shock protein 90kDa alpha (cytosolic). class B member 5 (pseudogene) |
| A_19_P00810365 | 9.54E-05 | 2.1193633 | up |  |  |  |
| A_23_P72387 | 0.00174388 | 2.113193 | up | AFAP1 | NM_001134647 | actin filament associated protein 1 |
| A_23_P24129 | 8.70E-04 | 2.1125982 | up | DKK1 | NM_012242 | dickkopf homolog 1 (Xenopus laevis) |
| A_33_P3702104 | 9.53E-07 | 2.1074965 | up | MGC34796 | BC034822 | SPR pseudogene |
| A_33_P3220827 | 2.85E-04 | 2.1066017 | up | PEA15 | NM_003768 | phosphoprotein enriched in astrocytes 15 |
| A_19_P00326488 | 1.43E-06 | 2.1046133 | up |  |  |  |
| A_33_P3384322 | 6.14E-04 | 2.1032565 | up | LOC100132006 | BX537921 | hypothetical protein LOC100132006 |
| A_33_P3411296 | 2.34E-04 | 2.0987713 | up | PURA | NM_005859 | purine-rich element binding protein A |
| A_23_P142849 | 0.01445046 | 2.0965018 | up | RND3 | NM_005168 | Rho family GTPase 3 |
| A_23_P145089 | 4.65E-05 | 2.0958548 | up | HSP90AB1 | NM_007355 | heat shock protein 90kDa alpha (cytosolic). class B member 1 |
| A_24_P38815 | 0.00185393 | 2.095182 | up | TPP1 | NM_000391 | tripeptidyl peptidase I |
| A_23_P87742 | 0.00417245 | 2.094016 | up | IFFO1 | NM_001039670 | intermediate filament family orphan 1 |
| A_33_P3347417 | 0.00106237 | 2.092435 | up | SPEN | NM_015001 | spen homolog. transcriptional regulator (Drosophila) |
| A_24_P336759 | 1.42E-04 | 2.0912743 | up | MCL1 | NM_021960 | myeloid cell leukemia sequence 1 (BCL2-related) |
| A_23_P360744 | 0.00108299 | 2.0883977 | up | RAG1 | NM_000448 | recombination activating gene 1 |
| A_24_P416177 | 0.00696005 | 2.0883436 | up | ADCY7 | NM_001114 | adenylate cyclase 7 |
| A_33_P3215277 | 1.45E-05 | 2.0882473 | up | TTBK2 | NM_173500 | tau tubulin kinase 2 |
| A_24_P66679 | 4.51E-05 | 2.0864522 | up | NAT12 | NM_001011713 | N-acetyltransferase 12 (GCN5-related. putative) |
| A_33_P3342111 | 2.03E-04 | 2.0857618 | up | ZNF169 | NM_194320 | zinc finger protein 169 |
| A_23_P15123 | 5.35E-06 | 2.07494 | up | UBFD1 | NM_019116 | ubiquitin family domain containing 1 |
| A_24_P137376 | 2.46E-05 | 2.0722504 | up | ATP2C1 | NM_001001485 | ATPase. Ca++ transporting. type 2C. member 1 |
| A_19_P00316844 | 0.0024284 | 2.0691526 | up |  |  |  |
| A_32_P67623 | 0.00758287 | 2.0653596 | up | FAM120C | NM_017848 | family with sequence similarity 120C |
| A_23_P11859 | 7.10E-04 | 2.0638378 | up | HSD17B7 | NM_016371 | hydroxysteroid (17-beta) dehydrogenase 7 |
| A_23_P99360 | 0.00279446 | 2.0630882 | up | TRIM13 | NM_213590 | tripartite motif-containing 13 |
| A_23_P16275 | 2.35E-04 | 2.0619602 | up | TSKS | NM_021733 | testis-specific serine kinase substrate |
| A_33_P3233580 | 2.07E-04 | 2.0616302 | up |  | BC098577 |  |
| A_23_P99693 | 2.76E-04 | 2.0588105 | up | ZBTB1 | NM_014950 | zinc finger and BTB domain containing 1 |
| A_33_P3397579 | 3.80E-04 | 2.0558197 | up |  | BC040611 |  |
| A_33_P3245183 | 0.00107225 | 2.0554955 | up | HRH1 | NM_001098213 | histamine receptor H1 |
| A_23_P133807 | 1.11E-04 | 2.0494313 | up | TAF8 | NM_138572 | TAF8 RNA polymerase II. TATA box binding protein (TBP)-associated factor. 43kDa |
| A_33_P3323742 | 5.00E-05 | 2.048094 | up | PNRC2 | NM_017761 | proline-rich nuclear receptor coactivator 2 |
| A_24_P175059 | 0.00216569 | 2.0446558 | up | ATG5 | NM_004849 | ATG5 autophagy related 5 homolog (S. cerevisiae) |
| A_33_P3361811 | 0.00413702 | 2.0441198 | up | NARG1L | NM_024561 | NMDA receptor regulated 1-like |
| A_33_P3318661 | 5.60E-05 | 2.0422125 | up | E2F7 | NM_203394 | E2F transcription factor 7 |
| A_33_P3225487 | 6.33E-04 | 2.0419762 | up | HSD17B7 | NM_016371 | hydroxysteroid (17-beta) dehydrogenase 7 |
| A_33_P3214597 | 4.97E-04 | 2.0405586 | up | RP9P | NR_003500 | retinitis pigmentosa 9 pseudogene |
| A_32_P3998 | 1.68E-04 | 2.0396903 | up | ZNF813 | NM_001004301 | zinc finger protein 813 |
| A_19_P00329121 | 8.25E-05 | 2.0389197 | up |  |  |  |
| A_24_P215475 | 0.00201147 | 2.0357025 | up | ZNF10 | NM_015394 | zinc finger protein 10 |
| A_23_P127652 | 2.89E-04 | 2.0349488 | up | ZNF202 | NM_003455 | zinc finger protein 202 |
| A_23_P47991 | 1.76E-04 | 2.0308528 | up | MED13L | NM_015335 | mediator complex subunit 13-like |
| A_33_P3260722 | 0.01097478 | 2.0291853 | up | UVRAG | NM_003369 | UV radiation resistance associated gene |
| A_24_P236522 | 1.86E-04 | 2.0290093 | up | CD2BP2 | NM_006110 | CD2 (cytoplasmic tail) binding protein 2 |
| A_24_P401090 | 0.00182952 | 2.0276625 | up |  |  |  |
| A_23_P122464 | 5.94E-04 | 2.020805 | up | ZNF193 | NM_006299 | zinc finger protein 193 |
| A_24_P345846 | 0.00140485 | 2.0200443 | up | ANTXR2 | NM_058172 | anthrax toxin receptor 2 |
| A_24_P410952 | 3.84E-04 | 2.0188932 | up | PEA15 | NM_003768 | phosphoprotein enriched in astrocytes 15 |
| A_23_P311201 | 2.51E-04 | 2.0176687 | up | SFRS13A |  | splicing factor. arginine/serine-rich 13A |
| A_19_P00808120 | 3.85E-05 | 2.0171518 | up |  |  |  |
| A_19_P00811226 | 3.81E-04 | 2.0143967 | up |  |  |  |
| A_33_P3285987 | 6.79E-05 | 2.0105844 | up | METT5D1 | NM_152636 | methyltransferase 5 domain containing 1 |
| A_24_P915692 | 5.52E-04 | 2.0102046 | up | PHLDA1 | NM_007350 | pleckstrin homology-like domain. family A. member 1 |
| A_23_P58579 | 3.93E-05 | 2.0078363 | up | TRIM52 | NM_032765 | tripartite motif-containing 52 |
| A_33_P3252925 | 3.03E-04 | 2.0049648 | up | PHF17 | NM_199320 | PHD finger protein 17 |
| A_23_P43425 | 9.27E-04 | 2.0019336 | up | C9orf40 | NM_017998 | chromosome 9 open reading frame 40 |
| ProbeName | p-value | FCAbsolute | regulation | GeneSymbol | GenbankAccession | GeneName |
| A_23_P501538 | 0.00223443 | 2.0010672 | down | HOXA3 | NM_153631 | homeobox A3 |
| A_23_P500956 | 9.47E-04 | 2.006008 | down | B3GNT2 | NM_006577 | UDP-GlcNAc:betaGal beta-1.3-N-acetylglucosaminyltransferase 2 |
| A_23_P10870 | 0.00270752 | 2.0064092 | down | DOLK | NM_014908 | dolichol kinase |
| A_23_P310350 | 3.90E-04 | 2.0065002 | down | SHPK | NM_013276 | sedoheptulokinase |
| A_23_P25626 | 0.00154062 | 2.0085366 | down | C13orf34 | NM_024808 | chromosome 13 open reading frame 34 |
| A_33_P3249434 | 6.52E-04 | 2.009228 | down | PLAA | NM_001031689 | phospholipase A2-activating protein |
| A_24_P139208 | 0.00266954 | 2.0099907 | down | USP25 | NM_013396 | ubiquitin specific peptidase 25 |
| A_23_P96325 | 0.00372003 | 2.0120335 | down | ERCC6L | NM_017669 | excision repair cross-complementing rodent repair deficiency. complementation group 6-like |
| A_24_P39101 | 1.05E-04 | 2.0125856 | down | KCTD10 | NM_031954 | potassium channel tetramerisation domain containing 10 |
| A_23_P35256 | 2.51E-04 | 2.0182514 | down | POLR3C | NM_006468 | polymerase (RNA) III (DNA directed) polypeptide C (62kD) |
| A_32_P38637 | 3.12E-05 | 2.0191529 | down | KRBA1 | NM_032534 | KRAB-A domain containing 1 |
| A_23_P74581 | 2.02E-05 | 2.019636 | down | SNHG12 | NR_024127 | small nucleolar RNA host gene 12 (non-protein coding) |
| A_33_P3242659 | 3.14E-04 | 2.0199037 | down | KIF13A | NM_022113 | kinesin family member 13A |
| A_23_P23616 | 0.00392646 | 2.021207 | down | PLEKHN1 | NM_032129 | pleckstrin homology domain containing. family N member 1 |
| A_24_P226278 | 0.0035797 | 2.0223045 | down | PHF15 | NM_015288 | PHD finger protein 15 |
| A_23_P61810 | 1.86E-04 | 2.022712 | down | BAIAP2 | NM_017450 | BAI1-associated protein 2 |
| A_23_P132388 | 0.00101121 | 2.0257318 | down | SCO2 | NM_005138 | SCO cytochrome oxidase deficient homolog 2 (yeast) |
| A_23_P24176 | 1.21E-05 | 2.0291433 | down | CCNJ | NM_019084 | cyclin J |
| A_23_P141345 | 7.71E-05 | 2.0297651 | down | MPP3 | NM_001932 | membrane protein. palmitoylated 3 (MAGUK p55 subfamily member 3) |
| A_32_P409222 | 9.67E-05 | 2.0306463 | down | ZNF628 | NM_033113 | zinc finger protein 628 |
| A_23_P349083 | 5.14E-04 | 2.03114 | down | FCHO2 | NM_138782 | FCH domain only 2 |
| A_24_P152404 | 4.29E-04 | 2.031551 | down |  | BC032118 |  |
| A_23_P36305 | 8.35E-04 | 2.031788 | down | ATG16L2 | NM_033388 | ATG16 autophagy related 16-like 2 (S. cerevisiae) |
| A_23_P122775 | 0.0012389 | 2.0362062 | down | RTN4IP1 | NM_032730 | reticulon 4 interacting protein 1 |
| A_33_P3262665 | 0.0012059 | 2.0368528 | down | MAP7D3 | NM_024597 | MAP7 domain containing 3 |
| A_33_P3396877 | 0.00169581 | 2.0369072 | down | RPP38 | NM_183005 | ribonuclease P/MRP 38kDa subunit |
| A_33_P3262012 | 0.00178324 | 2.0376668 | down |  |  |  |
| A_32_P92399 | 4.54E-04 | 2.0377915 | down | COG8 | NM_032382 | component of oligomeric golgi complex 8 |
| A_23_P314222 | 7.60E-04 | 2.0378938 | down | LEO1 | NM_138792 | Leo1. Paf1/RNA polymerase II complex component. homolog (S. cerevisiae) |
| A_23_P102925 | 3.38E-04 | 2.0382063 | down | PWP2 | NM_005049 | PWP2 periodic tryptophan protein homolog (yeast) |
| A_33_P3331345 | 6.55E-05 | 2.0387502 | down | DUSP11 | NM_003584 | dual specificity phosphatase 11 (RNA/RNP complex 1-interacting) |
| A_23_P111303 | 0.00140125 | 2.0396245 | down | RBM16 | NM_014892 | RNA binding motif protein 16 |
| A_23_P204375 | 6.18E-04 | 2.0397012 | down | LPAR5 | NM_020400 | lysophosphatidic acid receptor 5 |
| A_23_P15045 | 0.00207877 | 2.0398378 | down | E4F1 | NM_004424 | E4F transcription factor 1 |
| A_23_P207811 | 2.54E-04 | 2.040137 | down | PAIP1 | NM_006451 | poly(A) binding protein interacting protein 1 |
| A_24_P315444 | 1.15E-04 | 2.0404706 | down | LOC644422 | XR_019449 | similar to arginine/serine-rich splicing factor 6 |
| A_33_P3316493 | 0.00631891 | 2.0420477 | down | WDR91 | NM_014149 | WD repeat domain 91 |
| A_23_P62607 | 0.00934732 | 2.043391 | down | IL22RA1 | NM_021258 | interleukin 22 receptor. alpha 1 |
| A_24_P497226 | 2.57E-05 | 2.0454295 | down | RPS6KB1 | NM_003161 | ribosomal protein S6 kinase. 70kDa. polypeptide 1 |
| A_33_P3302632 | 0.006288 | 2.046736 | down | HIST1H2BE | NM_003523 | histone cluster 1. H2be |
| A_24_P162373 | 8.70E-04 | 2.0473454 | down | ZNRF3 | NM_032173 | zinc and ring finger 3 |
| A_23_P16058 | 0.00256864 | 2.0557334 | down | ZNF296 | NM_145288 | zinc finger protein 296 |
| A_23_P416434 | 0.00661134 | 2.055892 | down | PHF15 | NM_015288 | PHD finger protein 15 |
| A_33_P3357620 | 0.00168645 | 2.0569801 | down | C16orf53 | NM_024516 | chromosome 16 open reading frame 53 |
| A_24_P71700 | 5.13E-04 | 2.0572693 | down | ZBTB47 | NM_145166 | zinc finger and BTB domain containing 47 |
| A_23_P127948 | 4.85E-04 | 2.0573006 | down | ADM | NM_001124 | adrenomedullin |
| A_23_P129786 | 3.68E-05 | 2.0591867 | down | SREBF1 | NM_001005291 | sterol regulatory element binding transcription factor 1 |
| A_33_P3403778 | 6.30E-04 | 2.0593793 | down | ZNF579 | NM_152600 | zinc finger protein 579 |
| A_23_P152235 | 0.00722469 | 2.0602558 | down | IRX3 | NM_024336 | iroquois homeobox 3 |
| A_23_P110445 | 1.16E-04 | 2.0605965 | down | APBB3 | NM_006051 | amyloid beta (A4) precursor protein-binding. family B. member 3 |
| A_24_P294832 | 8.81E-04 | 2.061021 | down | PTP4A1 | NM_003463 | protein tyrosine phosphatase type IVA. member 1 |
| A_33_P3387045 | 9.65E-06 | 2.062652 | down | CYB5D1 | NM_144607 | cytochrome b5 domain containing 1 |
| A_24_P924862 | 3.35E-05 | 2.0628178 | down | RAPH1 | NM_213589 | Ras association (RalGDS/AF-6) and pleckstrin homology domains 1 |
| A_33_P3291349 | 9.52E-05 | 2.0629575 | down |  | CD237563 |  |
| A_33_P3220530 | 0.00154908 | 2.06345 | down | SFRS6 | NM_006275 | splicing factor. arginine/serine-rich 6 |
| A_24_P160466 | 0.00390758 | 2.0694907 | down | GPRIN1 | NM_052899 | G protein regulated inducer of neurite outgrowth 1 |
| A_33_P3238225 | 5.95E-04 | 2.0740998 | down |  |  |  |
| A_23_P89589 | 0.00650205 | 2.0743046 | down | PER1 | NM_002616 | period homolog 1 (Drosophila) |
| A_23_P209394 | 1.36E-04 | 2.0743356 | down | CFLAR | NM_001127184 | CASP8 and FADD-like apoptosis regulator |
| A_23_P345928 | 3.02E-04 | 2.0744264 | down | C12orf26 | NM_032230 | chromosome 12 open reading frame 26 |
| A_24_P302685 | 0.00913688 | 2.0744405 | down | ARHGEF4 | NM_015320 | Rho guanine nucleotide exchange factor (GEF) 4 |
| A_33_P3225522 | 0.00140204 | 2.0776446 | down | OAS2 | NM_001032731 | 2'-5'-oligoadenylate synthetase 2. 69/71kDa |
| A_24_P134653 | 2.24E-05 | 2.0783 | down | OFD1 | NM_003611 | oral-facial-digital syndrome 1 |
| A_23_P148919 | 0.00579085 | 2.0787077 | down | CPT2 | NM_000098 | carnitine palmitoyltransferase 2 |
| A_24_P225616 | 0.00205361 | 2.080086 | down | RRM2 | NM_001034 | ribonucleotide reductase M2 |
| A_23_P47790 | 8.07E-05 | 2.0802486 | down | METTL1 | NM_005371 | methyltransferase like 1 |
| A_23_P41344 | 0.00598089 | 2.0803652 | down | EREG | NM_001432 | epiregulin |
| A_23_P124927 | 0.00897783 | 2.0812685 | down | RGS14 | NM_006480 | regulator of G-protein signaling 14 |
| A_32_P12327 | 0.00630038 | 2.0819643 | down | LOC643783 | XM_931798 | hypothetical LOC643783 |
| A_23_P122532 | 4.57E-05 | 2.0873988 | down | C6orf47 | NM_021184 | chromosome 6 open reading frame 47 |
| A_23_P403521 | 0.00212795 | 2.0880961 | down | C7orf36 | NM_020192 | chromosome 7 open reading frame 36 |
| A_23_P78557 | 1.36E-05 | 2.0905724 | down | FBXL12 | NM_017703 | F-box and leucine-rich repeat protein 12 |
| A_23_P148484 | 5.18E-04 | 2.090969 | down | RLIM | NM_016120 | ring finger protein. LIM domain interacting |
| A_23_P92154 | 1.31E-04 | 2.0910225 | down | MBD4 | NM_003925 | methyl-CpG binding domain protein 4 |
| A_33_P3365357 | 8.71E-05 | 2.0952127 | down | HYAL2 | NM_003773 | hyaluronoglucosaminidase 2 |
| A_33_P3254801 | 0.00496334 | 2.095276 | down | OGFR | NM_007346 | opioid growth factor receptor |
| A_23_P35995 | 0.00206572 | 2.0959673 | down | ASAM | NM_024769 | adipocyte-specific adhesion molecule |
| A_23_P116387 | 0.00242584 | 2.0985577 | down | INCENP | NM_001040694 | inner centromere protein antigens 135/155kDa |
| A_33_P3268634 | 5.03E-05 | 2.0990026 | down | WIBG | NM_032345 | within bgcn homolog (Drosophila) |
| A_23_P91350 | 4.58E-04 | 2.1003776 | down | RP5-1022P6.2 | NM_019593 | hypothetical protein KIAA1434 |
| A_23_P432610 | 1.69E-05 | 2.1022968 | down | N4BP1 | NM_153029 | NEDD4 binding protein 1 |
| A_33_P3369761 | 0.0010766 | 2.102568 | down | PDP1 | NM_001161779 | pyruvate dehyrogenase phosphatase catalytic subunit 1 |
| A_23_P424878 | 0.00151682 | 2.1042733 | down | KIAA1688 | NM_025251 | KIAA1688 protein |
| A_23_P117734 | 2.38E-04 | 2.1044166 | down | LYSMD4 | NM_152449 | LysM. putative peptidoglycan-binding. domain containing 4 |
| A_23_P322 | 0.00170294 | 2.1070051 | down | EFNA4 | NM_182690 | ephrin-A4 |
| A_24_P230721 | 3.63E-05 | 2.1081278 | down | WDR73 | NM_032856 | WD repeat domain 73 |
| A_33_P3280945 | 4.81E-06 | 2.109587 | down | SNHG3 | NR_002909 | small nucleolar RNA host gene 3 (non-protein coding) |
| A_33_P3423949 | 0.0013526 | 2.1120675 | down | CBX2 | NM_005189 | chromobox homolog 2 (Pc class homolog. Drosophila) |
| A_23_P101111 | 1.05E-04 | 2.112183 | down | CTDP1 | NM_004715 | CTD (carboxy-terminal domain. RNA polymerase II. polypeptide A) phosphatase. subunit 1 |
| A_33_P3415663 | 0.00367038 | 2.112612 | down | MBLAC2 | NM_203406 | metallo-beta-lactamase domain containing 2 |
| A_33_P3357247 | 6.11E-04 | 2.114564 | down | USP36 | NM_025090 | ubiquitin specific peptidase 36 |
| A_33_P3815560 | 3.06E-04 | 2.1172726 | down | WDR6 | NM_018031 | WD repeat domain 6 |
| A_23_P48835 | 0.00103742 | 2.1181254 | down | KIF23 | NM_138555 | kinesin family member 23 |
| A_23_P366125 | 0.00655035 | 2.1186965 | down | EME2 | BC041011 | essential meiotic endonuclease 1 homolog 2 (S. pombe) |
| A_23_P142322 | 4.00E-04 | 2.119271 | down | CIRBP | NM_001280 | cold inducible RNA binding protein |
| A_33_P3384108 | 0.00612568 | 2.119982 | down | SLC19A1 | NM_194255 | solute carrier family 19 (folate transporter). member 1 |
| A_23_P80342 | 3.15E-04 | 2.1202364 | down | MAP3K7IP1 | NM_006116 | mitogen-activated protein kinase kinase kinase 7 interacting protein 1 |
| A_23_P20743 | 5.92E-05 | 2.1212788 | down | C9orf125 | NM_032342 | chromosome 9 open reading frame 125 |
| A_23_P159688 | 0.0010194 | 2.1224165 | down | TBC1D25 | NM_002536 | TBC1 domain family. member 25 |
| A_33_P3242649 | 0.00156329 | 2.1231549 | down | KIF18A | NM_031217 | kinesin family member 18A |
| A_32_P167122 | 0.00103853 | 2.1246998 | down | RCOR3 | NM_018254 | REST corepressor 3 |
| A_19_P00326178 | 0.0034395 | 2.1263795 | down |  |  |  |
| A_32_P142818 | 9.11E-04 | 2.1287458 | down | DLX1 | NM_178120 | distal-less homeobox 1 |
| A_23_P422193 | 0.001298 | 2.12898 | down | SUV39H1 | NM_003173 | suppressor of variegation 3-9 homolog 1 (Drosophila) |
| A_23_P157361 | 2.37E-04 | 2.1376965 | down | WDR60 | NM_018051 | WD repeat domain 60 |
| A_23_P214798 | 2.63E-05 | 2.1408856 | down | SYNCRIP | NM_006372 | synaptotagmin binding. cytoplasmic RNA interacting protein |
| A_33_P3221528 | 0.00104083 | 2.1415296 | down | IREB2 | NM_004136 | iron-responsive element binding protein 2 |
| A_24_P74070 | 2.74E-04 | 2.1438365 | down | PARD6G | NM_032510 | par-6 partitioning defective 6 homolog gamma (C. elegans) |
| A_32_P209230 | 0.00191039 | 2.146047 | down | CITED4 | NM_133467 | Cbp/p300-interacting transactivator. with Glu/Asp-rich carboxy-terminal domain. 4 |
| A_33_P3396956 | 0.00435772 | 2.1468449 | down | C1orf172 | NM_152365 | chromosome 1 open reading frame 172 |
| A_19_P00809039 | 0.01113736 | 2.146911 | down |  |  |  |
| A_23_P152356 | 0.00111758 | 2.1476493 | down | ZNF200 | NM_003454 | zinc finger protein 200 |
| A_23_P333705 | 0.00275238 | 2.14957 | down | NEK3 | NM_002498 | NIMA (never in mitosis gene a)-related kinase 3 |
| A_33_P3222045 | 3.53E-04 | 2.1503463 | down | FAM86A | NM_201400 | family with sequence similarity 86. member A |
| A_23_P150857 | 0.00185925 | 2.1519117 | down | SUOX | NM_000456 | sulfite oxidase |
| A_33_P3356320 | 0.00462467 | 2.15259 | down | C17orf97 | NM_001013672 | chromosome 17 open reading frame 97 |
| A_23_P32861 | 2.73E-04 | 2.1545808 | down | NMD3 | NM_015938 | NMD3 homolog (S. cerevisiae) |
| A_23_P166421 | 1.79E-04 | 2.1557329 | down | TBC1D10A | NM_031937 | TBC1 domain family. member 10A |
| A_32_P32739 | 1.85E-04 | 2.155922 | down | NAGS | NM_153006 | N-acetylglutamate synthase |
| A_23_P388812 | 0.005309 | 2.1573775 | down | CKAP2L | NM_152515 | cytoskeleton associated protein 2-like |
| A_33_P3375140 | 8.58E-05 | 2.1576881 | down | MCPH1 | NM_024596 | microcephalin 1 |
| A_23_P131676 | 0.00409525 | 2.1581025 | down | CXCR7 | NM_020311 | chemokine (C-X-C motif) receptor 7 |
| A_23_P50399 | 2.82E-05 | 2.169933 | down | DCAF15 | NM_138353 | DDB1 and CUL4 associated factor 15 |
| A_23_P3204 | 1.13E-05 | 2.169946 | down | MAPK6 | NM_002748 | mitogen-activated protein kinase 6 |
| A_23_P146765 | 0.00175679 | 2.1703658 | down | RNF139 | NM_007218 | ring finger protein 139 |
| A_33_P3362353 | 2.63E-04 | 2.1714013 | down |  | AB208831 |  |
| A_23_P376759 | 2.87E-04 | 2.1724627 | down | DUSP11 | NM_003584 | dual specificity phosphatase 11 (RNA/RNP complex 1-interacting) |
| A_23_P340922 | 9.00E-04 | 2.17461 | down | ZNF414 | NM_032370 | zinc finger protein 414 |
| A_23_P39185 | 9.54E-04 | 2.1753771 | down | RDH13 | NM_138412 | retinol dehydrogenase 13 (all-trans/9-cis) |
| A_33_P3331346 | 0.00134346 | 2.179573 | down | TRIM65 | NM_173547 | tripartite motif-containing 65 |
| A_23_P53588 | 7.61E-05 | 2.181965 | down | WNT5B | NM_030775 | wingless-type MMTV integration site family. member 5B |
| A_33_P3315355 | 6.17E-06 | 2.183135 | down | SETD6 | NM_001160305 | SET domain containing 6 |
| A_23_P75220 | 8.15E-05 | 2.183156 | down | SLC25A28 | NM_031212 | solute carrier family 25. member 28 |
| A_23_P423864 | 1.11E-04 | 2.1840582 | down | PHC2 | NM_198040 | polyhomeotic homolog 2 (Drosophila) |
| A_23_P571 | 3.08E-05 | 2.1848404 | down | SLC2A1 | NM_006516 | solute carrier family 2 (facilitated glucose transporter). member 1 |
| A_24_P226970 | 0.00285707 | 2.1852484 | down | ZNF365 | NM_014951 | zinc finger protein 365 |
| A_23_P77965 | 2.91E-04 | 2.185673 | down | HEATR6 | NM_022070 | HEAT repeat containing 6 |
| A_24_P418408 | 5.64E-04 | 2.186164 | down | FAM89A | NM_198552 | family with sequence similarity 89. member A |
| A_23_P48964 | 9.77E-05 | 2.1864953 | down | VPS33B | NM_018668 | vacuolar protein sorting 33 homolog B (yeast) |
| A_33_P3296707 | 3.06E-04 | 2.1865635 | down | FAM127C | NM_001078173 | family with sequence similarity 127. member C |
| A_33_P3402035 | 4.48E-05 | 2.1869488 | down | TRAF4 | NM_004295 | TNF receptor-associated factor 4 |
| A_33_P3240053 | 3.17E-04 | 2.1886349 | down | UFSP1 | NM_001015072 | UFM1-specific peptidase 1 (non-functional) |
| A_33_P3383866 | 0.00671447 | 2.1886954 | down | TREX1 | NM_016381 | three prime repair exonuclease 1 |
| A_23_P168951 | 4.21E-04 | 2.190618 | down | ZHX2 | NM_014943 | zinc fingers and homeoboxes 2 |
| A_23_P79221 | 8.89E-05 | 2.194269 | down | ACVR1 | NM_001105 | activin A receptor. type I |
| A_24_P48898 | 7.91E-05 | 2.196922 | down | APOL2 | NM_145637 | apolipoprotein L. 2 |
| A_23_P210747 | 3.06E-04 | 2.1985853 | down | TRMT6 | NM_015939 | tRNA methyltransferase 6 homolog (S. cerevisiae) |
| A_23_P99747 | 0.00706585 | 2.1988602 | down | CDKL1 | NM_004196 | cyclin-dependent kinase-like 1 (CDC2-related kinase) |
| A_19_P00321073 | 1.13E-05 | 2.1990857 | down |  |  |  |
| A_33_P3423820 | 1.14E-04 | 2.2038515 | down | ZC3H3 | NM_015117 | zinc finger CCCH-type containing 3 |
| A_19_P00319907 | 6.03E-04 | 2.204345 | down |  |  |  |
| A_23_P21324 | 0.01113742 | 2.2050028 | down | TWIST2 | NM_057179 | twist homolog 2 (Drosophila) |
| A_32_P131031 | 0.00393199 | 2.2052946 | down | MACC1 | NM_182762 | metastasis associated in colon cancer 1 |
| A_32_P47643 | 0.00373733 | 2.2073433 | down | FAM110C | CR601458 | family with sequence similarity 110. member C |
| A_33_P3311755 | 3.10E-04 | 2.2081714 | down | KIF23 | NM_138555 | kinesin family member 23 |
| A_23_P53856 | 1.58E-04 | 2.210343 | down | N4BP2L2 | NM_014887 | NEDD4 binding protein 2-like 2 |
| A_23_P152651 | 7.76E-05 | 2.211648 | down | DDX42 | NM_007372 | DEAD (Asp-Glu-Ala-Asp) box polypeptide 42 |
| A_23_P169470 | 8.72E-05 | 2.21568 | down | CDK9 | NM_001261 | cyclin-dependent kinase 9 |
| A_24_P390403 | 1.88E-04 | 2.217264 | down | RTF1 | NM_015138 | Rtf1. Paf1/RNA polymerase II complex component. homolog (S. cerevisiae) |
| A_33_P3226039 | 4.26E-05 | 2.2188537 | down |  | XM_002342244 |  |
| A_33_P3256858 | 0.00720862 | 2.2202554 | down | C14orf80 | NM_001134875 | chromosome 14 open reading frame 80 |
| A_23_P107744 | 0.01315364 | 2.2212327 | down | S1PR5 | NM_030760 | sphingosine-1-phosphate receptor 5 |
| A_24_P155791 | 0.00309482 | 2.2228696 | down | ASB7 | NM_024708 | ankyrin repeat and SOCS box-containing 7 |
| A_23_P88184 | 3.91E-05 | 2.2249498 | down | BTBD7 | NM_018167 | BTB (POZ) domain containing 7 |
| A_23_P150693 | 0.00173299 | 2.2260492 | down | FJX1 | NM_014344 | four jointed box 1 (Drosophila) |
| A_23_P137484 | 0.00143511 | 2.2290177 | down | L1TD1 | NM_019079 | LINE-1 type transposase domain containing 1 |
| A_19_P00317260 | 4.52E-05 | 2.2300243 | down |  |  |  |
| A_33_P3364051 | 6.34E-04 | 2.2307043 | down | TOP3B | NM_003935 | topoisomerase (DNA) III beta |
| A_19_P00811456 | 0.0022144 | 2.231174 | down |  |  |  |
| A_23_P215132 | 0.00482345 | 2.2319527 | down | WDR91 | NM_014149 | WD repeat domain 91 |
| A_24_P373152 | 5.44E-05 | 2.2345736 | down | CFL2 | NM_021914 | cofilin 2 (muscle) |
| A_23_P12336 | 0.00154983 | 2.2347066 | down | PRMT6 | NM_018137 | protein arginine methyltransferase 6 |
| A_23_P218282 | 1.53E-04 | 2.2400377 | down | ZNF434 | NM_017810 | zinc finger protein 434 |
| A_33_P3400374 | 3.93E-04 | 2.2449977 | down | PRIC285 | NM_001037335 | peroxisomal proliferator-activated receptor A interacting complex 285 |
| A_23_P33809 | 0.00196897 | 2.247227 | down | IMP3 | NM_018285 | IMP3. U3 small nucleolar ribonucleoprotein. homolog (yeast) |
| A_33_P3360665 | 1.56E-05 | 2.249215 | down | ACVR1 | NM_001105 | activin A receptor. type I |
| A_23_P156355 | 2.83E-05 | 2.2516894 | down | TMEM161B | NM_153354 | transmembrane protein 161B |
| A_23_P434890 | 4.53E-05 | 2.2517517 | down | CARD10 | NM_014550 | caspase recruitment domain family. member 10 |
| A_33_P3258346 | 0.0012275 | 2.2522564 | down | XAF1 | NM_017523 | XIAP associated factor 1 |
| A_33_P3421200 | 5.00E-04 | 2.252292 | down | hCG_1990547 | NR_024361 | family with sequence similarity 86. member A pseudogene |
| A_33_P3283196 | 2.52E-05 | 2.253036 | down |  |  |  |
| A_23_P160809 | 9.31E-05 | 2.253542 | down | COG2 | NM_007357 | component of oligomeric golgi complex 2 |
| A_23_P142714 | 1.94E-04 | 2.256609 | down | SLC25A12 | NM_003705 | solute carrier family 25 (mitochondrial carrier. Aralar). member 12 |
| A_23_P34510 | 7.80E-04 | 2.258187 | down | PHC2 | NM_198040 | polyhomeotic homolog 2 (Drosophila) |
| A_33_P3328928 | 1.72E-04 | 2.2582216 | down | BMP2K | NM_017593 | BMP2 inducible kinase |
| A_33_P3224705 | 4.50E-05 | 2.2589886 | down |  |  |  |
| A_23_P122624 | 3.50E-04 | 2.259152 | down | LTV1 | NM_032860 | LTV1 homolog (S. cerevisiae) |
| A_23_P38677 | 2.98E-04 | 2.259906 | down | SLMO1 | NM_006553 | slowmo homolog 1 (Drosophila) |
| A_24_P49190 | 1.09E-05 | 2.2619658 | down | C17orf58 | NM_181655 | chromosome 17 open reading frame 58 |
| A_23_P367676 | 0.0018971 | 2.2636726 | down | SIN3A | NM_015477 | SIN3 homolog A. transcription regulator (yeast) |
| A_23_P365418 | 9.14E-04 | 2.266255 | down |  | XM_002346055 |  |
| A_23_P160537 | 0.00201974 | 2.2663486 | down | C1orf135 | NM_024037 | chromosome 1 open reading frame 135 |
| A_23_P94762 | 0.00458316 | 2.2687545 | down | ZNF354B | NM_058230 | zinc finger protein 354B |
| A_23_P361841 | 2.56E-04 | 2.2695873 | down | RIMKLB | NM_020734 | ribosomal modification protein rimK-like family member B |
| A_23_P316460 | 2.38E-04 | 2.2718647 | down | C7orf29 | NM_138434 | chromosome 7 open reading frame 29 |
| A_19_P00315755 | 7.72E-04 | 2.2737646 | down |  |  |  |
| A_23_P334282 | 1.61E-04 | 2.2742064 | down | BMP2K | NM_017593 | BMP2 inducible kinase |
| A_33_P3321205 | 1.23E-04 | 2.2742333 | down | BEGAIN | NM_001159531 | brain-enriched guanylate kinase-associated homolog (rat) |
| A_24_P750305 | 6.05E-04 | 2.2810585 | down | LOC643837 | NR_015368 | hypothetical LOC643837 |
| A_24_P88554 | 9.91E-05 | 2.282178 | down | PEX11B | NM_003846 | peroxisomal biogenesis factor 11 beta |
| A_24_P36745 | 8.63E-04 | 2.2826967 | down | CXorf38 | NM_144970 | chromosome X open reading frame 38 |
| A_33_P3209096 | 2.51E-07 | 2.2861705 | down | CD58 | NM_001779 | CD58 molecule |
| A_23_P102242 | 1.88E-04 | 2.2864757 | down | MARCH7 | NM_022826 | membrane-associated ring finger (C3HC4) 7 |
| A_23_P162142 | 0.0012176 | 2.2870314 | down | TSKU | NM_015516 | tsukushin |
| A_33_P3309859 | 0.00115198 | 2.2899263 | down | LOC100132805 | XM_001719188 | hypothetical LOC100132805 |
| A_23_P7976 | 0.00463658 | 2.2915432 | down | HIST1H1E | NM_005321 | histone cluster 1. H1e |
| A_23_P47004 | 4.89E-05 | 2.2954412 | down | DHX32 | NM_018180 | DEAH (Asp-Glu-Ala-His) box polypeptide 32 |
| A_23_P356122 | 2.95E-05 | 2.2964885 | down | ZNF451 | NM_001031623 | zinc finger protein 451 |
| A_23_P81121 | 0.00433713 | 2.2999907 | down | EXOSC9 | NM_005033 | exosome component 9 |
| A_23_P38408 | 1.66E-04 | 2.3011107 | down | GGNBP2 | NM_024835 | gametogenetin binding protein 2 |
| A_33_P3336103 | 5.08E-04 | 2.3020613 | down | TIGD5 | NM_032862 | tigger transposable element derived 5 |
| A_19_P00319646 | 4.38E-05 | 2.3120582 | down |  |  |  |
| A_23_P28730 | 7.30E-05 | 2.3128865 | down | ZNF512B | NM_020713 | zinc finger protein 512B |
| A_24_P117323 | 3.72E-04 | 2.3156419 | down | KLHL22 | NM_032775 | kelch-like 22 (Drosophila) |
| A_23_P121222 | 0.00158737 | 2.3191712 | down | RAD18 | NM_020165 | RAD18 homolog (S. cerevisiae) |
| A_32_P46840 | 9.73E-05 | 2.3196902 | down | LOC729680 | XR_040503 | hypothetical protein LOC729680 |
| A_24_P49447 | 6.30E-04 | 2.319953 | down | KIAA1432 | NM_020829 | KIAA1432 |
| A_33_P3416286 | 4.30E-04 | 2.3211207 | down | RBM12 | NM_006047 | RNA binding motif protein 12 |
| A_23_P431381 | 0.00373543 | 2.3223937 | down | C14orf80 | NM_001134875 | chromosome 14 open reading frame 80 |
| A_23_P366682 | 9.62E-05 | 2.3257506 | down | C19orf20 | NM_033513 | chromosome 19 open reading frame 20 |
| A_23_P395426 | 0.00163498 | 2.3267963 | down | DIDO1 | NM_022105 | death inducer-obliterator 1 |
| A_33_P3229241 | 0.00239998 | 2.332011 | down | HIST2H2BF | NM_001024599 | histone cluster 2. H2bf |
| A_23_P145114 | 0.00191909 | 2.3321924 | down | GCLC | NM_001498 | glutamate-cysteine ligase. catalytic subunit |
| A_23_P323943 | 5.72E-04 | 2.333175 | down | SLC5A12 | NM_178498 | solute carrier family 5 (sodium/glucose cotransporter). member 12 |
| A_23_P334955 | 3.00E-05 | 2.3372536 | down | FAM167A | NM_053279 | family with sequence similarity 167. member A |
| A_23_P101380 | 4.64E-06 | 2.3415654 | down | B3GNT8 | NM_198540 | UDP-GlcNAc:betaGal beta-1.3-N-acetylglucosaminyltransferase 8 |
| A_33_P3411357 | 3.72E-05 | 2.3429184 | down | DGCR11 | NR_024157 | DiGeorge syndrome critical region gene 11 |
| A_24_P141736 | 3.93E-05 | 2.3439026 | down | METAP2 | NM_006838 | methionyl aminopeptidase 2 |
| A_23_P308150 | 0.00464441 | 2.3454988 | down | FAM123B | NM_152424 | family with sequence similarity 123B |
| A_23_P54556 | 1.90E-05 | 2.3458622 | down | MKL2 | NM_014048 | MKL/myocardin-like 2 |
| A_33_P3240702 | 4.41E-04 | 2.346878 | down | RBBP8 | NM_002894 | retinoblastoma binding protein 8 |
| A_33_P3254460 | 8.43E-04 | 2.3475034 | down | DLK2 | NM_206539 | delta-like 2 homolog (Drosophila) |
| A_24_P305933 | 8.65E-04 | 2.3500504 | down | TMCC3 | NM_020698 | transmembrane and coiled-coil domain family 3 |
| A_23_P4679 | 0.00320682 | 2.3503318 | down | ERF | NM_006494 | Ets2 repressor factor |
| A_23_P406616 | 1.10E-04 | 2.3550386 | down | FLJ36031 | NM_175884 | hypothetical protein FLJ36031 |
| A_23_P50000 | 1.64E-06 | 2.3591 | down | FAM57A | NM_024792 | family with sequence similarity 57. member A |
| A_24_P333663 | 2.27E-05 | 2.3607166 | down | MAPK6 | NM_002748 | mitogen-activated protein kinase 6 |
| A_23_P419202 | 3.58E-04 | 2.3617928 | down | ZNF658 | NM_033160 | zinc finger protein 658 |
| A_24_P23034 | 0.00203701 | 2.3619123 | down | ZNFX1 | NM_021035 | zinc finger. NFX1-type containing 1 |
| A_24_P69439 | 3.65E-04 | 2.366074 | down | SLC25A32 | NM_030780 | solute carrier family 25. member 32 |
| A_33_P3296308 | 3.19E-04 | 2.3667698 | down | DCAF16 | NM_017741 | DDB1 and CUL4 associated factor 16 |
| A_23_P314086 | 7.13E-04 | 2.3673806 | down | RNF126 | NM_194460 | ring finger protein 126 |
| A_23_P47116 | 3.44E-04 | 2.370476 | down | RASSF7 | NM_003475 | Ras association (RalGDS/AF-6) domain family (N-terminal) member 7 |
| A_23_P395595 | 4.66E-04 | 2.3715892 | down | FNBP4 | NM_015308 | formin binding protein 4 |
| A_24_P318897 | 7.60E-05 | 2.3720715 | down | SNX21 | NM_001042633 | sorting nexin family member 21 |
| A_33_P3352782 | 4.98E-04 | 2.3807116 | down | TMCO7 | NM_024562 | transmembrane and coiled-coil domains 7 |
| A_33_P3338724 | 4.26E-04 | 2.3810003 | down | LENG9 | NM_198988 | leukocyte receptor cluster (LRC) member 9 |
| A_24_P381199 | 6.29E-04 | 2.3874311 | down | TRIM6 | NM_001003818 | tripartite motif-containing 6 |
| A_33_P3289356 | 2.73E-06 | 2.3881073 | down | CD58 | NM_001779 | CD58 molecule |
| A_33_P3407524 | 0.00288619 | 2.405087 | down | SUV39H1 | NM_003173 | suppressor of variegation 3-9 homolog 1 (Drosophila) |
| A_33_P3288364 | 0.00207668 | 2.4076242 | down | SPATA2L | NM_152339 | spermatogenesis associated 2-like |
| A_23_P304716 | 0.00639207 | 2.4086957 | down | HES2 | NM_019089 | hairy and enhancer of split 2 (Drosophila) |
| A_23_P380857 | 3.67E-04 | 2.4111688 | down | APOL4 | NM_030643 | apolipoprotein L. 4 |
| A_23_P218654 | 6.67E-05 | 2.413411 | down | ZGPAT | NM_032527 | zinc finger. CCCH-type with G patch domain |
| A_23_P304897 | 0.00262612 | 2.422637 | down | BDKRB2 | NM_000623 | bradykinin receptor B2 |
| A_23_P129569 | 1.03E-04 | 2.424489 | down | PALB2 | NM_024675 | partner and localizer of BRCA2 |
| A_23_P502832 | 0.00373033 | 2.4274626 | down | RBM12 | NM_006047 | RNA binding motif protein 12 |
| A_23_P116602 | 1.53E-04 | 2.4275308 | down | USP35 | NM_020798 | ubiquitin specific peptidase 35 |
| A_23_P100220 | 2.60E-06 | 2.444663 | down | ESRP2 | NM_024939 | epithelial splicing regulatory protein 2 |
| A_33_P3419234 | 6.13E-04 | 2.4474056 | down | DCAF4 | NM_015604 | DDB1 and CUL4 associated factor 4 |
| A_23_P320457 | 1.03E-05 | 2.4488025 | down | FAM40A | NM_033088 | family with sequence similarity 40. member A |
| A_23_P142974 | 5.68E-04 | 2.4593756 | down | ARHGAP25 | NM_001007231 | Rho GTPase activating protein 25 |
| A_23_P10156 | 1.87E-04 | 2.4599006 | down | CHMP6 | NM_024591 | chromatin modifying protein 6 |
| A_23_P99917 | 1.33E-05 | 2.4755352 | down | WDR73 | NM_032856 | WD repeat domain 73 |
| A_23_P53126 | 3.23E-04 | 2.4764123 | down | LMO2 | NM_005574 | LIM domain only 2 (rhombotin-like 1) |
| A_23_P214300 | 0.00172788 | 2.4767015 | down | GSTA2 | NM_000846 | glutathione S-transferase alpha 2 |
| A_23_P433791 | 1.01E-05 | 2.4938183 | down | C1orf124 | NM_001010984 | chromosome 1 open reading frame 124 |
| A_33_P3241482 | 1.98E-05 | 2.503342 | down | ZNF346 | NM_012279 | zinc finger protein 346 |
| A_24_P48248 | 8.03E-04 | 2.5062258 | down | C17orf53 | NM_024032 | chromosome 17 open reading frame 53 |
| A_23_P149626 | 3.27E-05 | 2.508419 | down | PLEKHG5 | NM_198681 | pleckstrin homology domain containing. family G (with RhoGef domain) member 5 |
| A_23_P376870 | 7.39E-04 | 2.5101068 | down | C14orf79 | NM_174891 | chromosome 14 open reading frame 79 |
| A_23_P364792 | 9.38E-04 | 2.5170674 | down | CYorf15A | NM_001005852 | chromosome Y open reading frame 15A |
| A_23_P44363 | 0.00105638 | 2.518409 | down | CASKIN2 | NM_020753 | CASK interacting protein 2 |
| A_24_P278299 | 1.42E-04 | 2.521272 | down | ASB13 | NM_024701 | ankyrin repeat and SOCS box-containing 13 |
| A_24_P928969 | 9.64E-05 | 2.5237398 | down | PTPN3 | NM_001145369 | protein tyrosine phosphatase. non-receptor type 3 |
| A_23_P217899 | 5.61E-04 | 2.524478 | down | CCNL2 | NM_001039577 | cyclin L2 |
| A_24_P133488 | 0.00190134 | 2.5271847 | down | CDCA4 | NM_017955 | cell division cycle associated 4 |
| A_23_P212617 | 2.99E-05 | 2.528552 | down | TFRC | NM_003234 | transferrin receptor (p90. CD71) |
| A_23_P34568 | 3.44E-04 | 2.528993 | down | ADPRHL2 | NM_017825 | ADP-ribosylhydrolase like 2 |
| A_23_P321473 | 2.92E-04 | 2.5296266 | down | FAM76A | NM_152660 | family with sequence similarity 76. member A |
| A_23_P158880 | 5.83E-05 | 2.531339 | down | STARD5 | NM_181900 | StAR-related lipid transfer (START) domain containing 5 |
| A_23_P360626 | 0.00142348 | 2.5377655 | down | PLD6 | NM_178836 | phospholipase D family. member 6 |
| A_24_P416961 | 1.68E-04 | 2.541495 | down | ARVCF | NM_001670 | armadillo repeat gene deletes in velocardiofacial syndrome |
| A_23_P424582 | 4.33E-07 | 2.5540974 | down | EGFL8 | NM_030652 | EGF-like-domain. multiple 8 |
| A_19_P00810040 | 4.20E-04 | 2.5562465 | down |  |  |  |
| A_24_P241318 | 7.18E-04 | 2.5568495 | down | DCAF4 | NM_181340 | DDB1 and CUL4 associated factor 4 |
| A_23_P358944 | 5.25E-04 | 2.559567 | down | PML | NM_033244 | promyelocytic leukemia |
| A_23_P85188 | 2.40E-05 | 2.561103 | down | ARMCX5 | NM_022838 | armadillo repeat containing. X-linked 5 |
| A_32_P220739 | 0.00160951 | 2.5619276 | down | ZNF658 | NM_033160 | zinc finger protein 658 |
| A_23_P403284 | 0.00347674 | 2.5670056 | down | OTX1 | NM_014562 | orthodenticle homeobox 1 |
| A_19_P00331841 | 4.80E-04 | 2.5689845 | down |  |  |  |
| A_23_P108437 | 1.20E-04 | 2.57905 | down | FZD5 | NM_003468 | frizzled homolog 5 (Drosophila) |
| A_23_P154585 | 4.89E-04 | 2.5803006 | down | SNX21 | NM_001042633 | sorting nexin family member 21 |
| A_23_P159101 | 1.98E-04 | 2.581681 | down | SNRNP35 | NM_022717 | small nuclear ribonucleoprotein 35kDa (U11/U12) |
| A_24_P198820 | 2.99E-05 | 2.5820541 | down | CRKRS | NM_016507 | Cdc2-related kinase. arginine/serine-rich |
| A_33_P3272231 | 1.96E-04 | 2.5882285 | down | MFSD2A | NM_001136493 | major facilitator superfamily domain containing 2A |
| A_24_P272225 | 1.15E-05 | 2.5952413 | down | LOC645676 | NR_027023 | hypothetical LOC645676 |
| A_23_P99930 | 7.18E-04 | 2.6036694 | down | TIPIN | NM_017858 | TIMELESS interacting protein |
| A_33_P3413845 | 8.15E-04 | 2.6199913 | down | TIMM13 | NM_012458 | translocase of inner mitochondrial membrane 13 homolog (yeast) |
| A_23_P418234 | 2.12E-04 | 2.6212478 | down | PHLPP2 | NM_015020 | PH domain and leucine rich repeat protein phosphatase 2 |
| A_33_P3393796 | 2.95E-04 | 2.622745 | down | TRMT5 | NM_020810 | TRM5 tRNA methyltransferase 5 homolog (S. cerevisiae) |
| A_33_P3288219 | 8.74E-06 | 2.6288133 | down |  |  |  |
| A_23_P8380 | 1.30E-04 | 2.63156 | down | C7orf49 | NM_024033 | chromosome 7 open reading frame 49 |
| A_23_P120056 | 1.20E-05 | 2.6404328 | down | RTKN | NM_033046 | rhotekin |
| A_33_P3300757 | 1.21E-04 | 2.64303 | down | DOPEY1 | NM_015018 | dopey family member 1 |
| A_24_P377225 | 1.99E-04 | 2.6453578 | down | USP46 | NM_022832 | ubiquitin specific peptidase 46 |
| A_23_P99632 | 1.38E-04 | 2.6554801 | down | RNF31 | NM_017999 | ring finger protein 31 |
| A_23_P69452 | 4.48E-04 | 2.658059 | down | DBR1 | NM_016216 | debranching enzyme homolog 1 (S. cerevisiae) |
| A_23_P314712 | 0.00100404 | 2.658853 | down | CABYR | NM_012189 | calcium binding tyrosine-(Y)-phosphorylation regulated |
| A_33_P3349646 | 3.78E-04 | 2.6591616 | down | PCDH7 | NM_002589 | protocadherin 7 |
| A_24_P28657 | 4.69E-05 | 2.6604261 | down | AHCTF1 | NM_015446 | AT hook containing transcription factor 1 |
| A_23_P23815 | 7.66E-04 | 2.6645093 | down | SLC30A1 | NM_021194 | solute carrier family 30 (zinc transporter). member 1 |
| A_33_P3289128 | 2.09E-04 | 2.6664977 | down | ZBTB42 | NM_001137601 | zinc finger and BTB domain containing 42 |
| A_23_P28953 | 0.00149738 | 2.6718152 | down | DNMT3B | NM_175850 | DNA (cytosine-5-)-methyltransferase 3 beta |
| A_23_P24922 | 4.36E-04 | 2.678986 | down | LIPT2 | NM_001144869 | lipoyl(octanoyl) transferase 2 (putative) |
| A_23_P2271 | 7.68E-06 | 2.6797764 | down | PTHLH | NM_198965 | parathyroid hormone-like hormone |
| A_23_P86917 | 4.52E-04 | 2.6827822 | down | FADD | NM_003824 | Fas (TNFRSF6)-associated via death domain |
| A_33_P3296230 | 8.09E-05 | 2.6882937 | down |  |  |  |
| A_24_P159227 | 7.62E-04 | 2.6891718 | down | PAK6 | NM_020168 | p21 protein (Cdc42/Rac)-activated kinase 6 |
| A_23_P383278 | 1.97E-04 | 2.706995 | down | PYCRL | NM_023078 | pyrroline-5-carboxylate reductase-like |
| A_23_P55256 | 3.65E-04 | 2.7070992 | down | ZNF652 | NM_014897 | zinc finger protein 652 |
| A_23_P123454 | 0.00142788 | 2.7223406 | down | NUDT18 | NM_024815 | nudix (nucleoside diphosphate linked moiety X)-type motif 18 |
| A_24_P557479 | 0.00120372 | 2.7240822 | down | XAF1 | NM_017523 | XIAP associated factor 1 |
| A_33_P3411427 | 1.48E-05 | 2.7291145 | down | ZNF837 | NM_001129730 | zinc finger protein 837 |
| A_23_P132159 | 1.78E-04 | 2.7305243 | down | USP18 | NM_017414 | ubiquitin specific peptidase 18 |
| A_23_P501770 | 1.71E-04 | 2.7412095 | down | ATRIP | NM_032166 | ATR interacting protein |
| A_24_P395621 | 7.73E-04 | 2.755726 | down | RUNDC1 | NM_173079 | RUN domain containing 1 |
| A_33_P3260455 | 2.88E-04 | 2.758407 | down |  |  |  |
| A_33_P3212570 | 0.00101646 | 2.7714612 | down |  |  |  |
| A_23_P75071 | 8.18E-05 | 2.7828562 | down | KIF20B | NM_016195 | kinesin family member 20B |
| A_33_P3339336 | 8.15E-04 | 2.8012726 | down | LOC653113 | NR_024254 | family with sequence similarity 86. member A pseudogene |
| A_24_P79241 | 1.74E-04 | 2.8224747 | down | UBOX5 | NM_014948 | U-box domain containing 5 |
| A_23_P63153 | 3.85E-04 | 2.8326595 | down | DDX20 | NM_007204 | DEAD (Asp-Glu-Ala-Asp) box polypeptide 20 |
| A_32_P514790 | 1.67E-04 | 2.8479238 | down | UNK | NM_001080419 | unkempt homolog (Drosophila) |
| A_23_P45087 | 4.28E-04 | 2.8633912 | down | ZNF107 | NM_016220 | zinc finger protein 107 |
| A_24_P139191 | 1.16E-04 | 2.8843176 | down | ITCH | NM_031483 | itchy E3 ubiquitin protein ligase homolog (mouse) |
| A_23_P70566 | 9.48E-05 | 2.915228 | down | FKBPL | NM_022110 | FK506 binding protein like |
| A_23_P432360 | 8.67E-06 | 2.9454195 | down | PMM2 | NM_000303 | phosphomannomutase 2 |
| A_33_P3348802 | 4.29E-05 | 2.984326 | down | LOC729570 | XM_001717053 | hypothetical LOC729570 |
| A_23_P58489 | 1.62E-04 | 2.990936 | down | FASTKD3 | NM_024091 | FAST kinase domains 3 |
| A_23_P20722 | 7.91E-04 | 2.9972143 | down | SNAPC4 | NM_003086 | small nuclear RNA activating complex. polypeptide 4. 190kDa |
| A_33_P3313245 | 7.40E-05 | 3.001764 | down | AMACR | NM_014324 | alpha-methylacyl-CoA racemase |
| A_23_P91390 | 7.82E-04 | 3.0096686 | down | THBD | NM_000361 | thrombomodulin |
| A_33_P3356711 | 5.32E-05 | 3.0138175 | down | ING3 | NM_198267 | inhibitor of growth family. member 3 |
| A_32_P76720 | 5.66E-05 | 3.014938 | down | NT5DC3 | NM_001031701 | 5'-nucleotidase domain containing 3 |
| A_32_P54553 | 1.60E-04 | 3.0166538 | down | USP41 | XM_937988 | ubiquitin specific peptidase 41 |
| A_32_P116660 | 9.00E-04 | 3.0573792 | down | RBM43 | NM_198557 | RNA binding motif protein 43 |
| A_33_P3267502 | 7.67E-04 | 3.090162 | down | ANKRD16 | NM_001009941 | ankyrin repeat domain 16 |
| A_23_P2705 | 2.01E-04 | 3.0928633 | down | LPAR6 | NM_005767 | lysophosphatidic acid receptor 6 |
| A_24_P129277 | 2.71E-04 | 3.1075318 | down | NOD1 | NM_006092 | nucleotide-binding oligomerization domain containing 1 |
| A_23_P113825 | 4.41E-05 | 3.1113112 | down | NACC2 | NM_144653 | NACC family member 2. BEN and BTB (POZ) domain containing |
| A_24_P30194 | 0.00134731 | 3.1377583 | down | IFIT5 | NM_012420 | interferon-induced protein with tetratricopeptide repeats 5 |
| A_23_P377819 | 1.37E-04 | 3.143879 | down | SFRS5 | NM_001039465 | splicing factor. arginine/serine-rich 5 |
| A_24_P298013 | 1.43E-05 | 3.1455116 | down | GTPBP2 | NM_019096 | GTP binding protein 2 |
| A_23_P204579 | 3.46E-06 | 3.1632054 | down | TDG | NM_003211 | thymine-DNA glycosylase |
| A_33_P3376636 | 1.52E-04 | 3.1646976 | down | OPA3 | NM_001017989 | optic atrophy 3 (autosomal recessive. with chorea and spastic paraplegia) |
| A_23_P310483 | 1.53E-04 | 3.2051096 | down | C8orf58 | NM_001013842 | chromosome 8 open reading frame 58 |
| A_23_P11729 | 2.36E-04 | 3.209637 | down | ZBTB7B | NM_015872 | zinc finger and BTB domain containing 7B |
| A_33_P3402020 | 0.00181768 | 3.2123008 | down | CCDC8 | NM_032040 | coiled-coil domain containing 8 |
| A_23_P413803 | 8.88E-05 | 3.2243416 | down | C5orf37 | NM_152408 | chromosome 5 open reading frame 37 |
| A_23_P74449 | 9.95E-04 | 3.2635815 | down | HPDL | NM_032756 | 4-hydroxyphenylpyruvate dioxygenase-like |
| A_33_P3228722 | 9.44E-05 | 3.2888496 | down |  | AL096745 |  |
| A_24_P371425 | 1.14E-04 | 3.2896223 | down | ATRIP | NM_032166 | ATR interacting protein |
| A_23_P204947 | 4.79E-04 | 3.3055377 | down | GJB2 | NM_004004 | gap junction protein. beta 2. 26kDa |
| A_33_P3372910 | 6.32E-05 | 3.3081958 | down | DDX58 | NM_014314 | DEAD (Asp-Glu-Ala-Asp) box polypeptide 58 |
| A_23_P108501 | 0.00103031 | 3.3243678 | down | EPHA4 | NM_004438 | EPH receptor A4 |
| A_33_P3366146 | 1.32E-04 | 3.3425748 | down | SFRS5 | NM_001039465 | splicing factor. arginine/serine-rich 5 |
| A_24_P83379 | 4.35E-04 | 3.3460162 | down | WDFY3 | NM_178583 | WD repeat and FYVE domain containing 3 |
| A_23_P106633 | 3.73E-04 | 3.3551044 | down | DDX28 | NM_018380 | DEAD (Asp-Glu-Ala-Asp) box polypeptide 28 |
| A_33_P3357445 | 8.47E-06 | 3.3614013 | down | TDG | NM_003211 | thymine-DNA glycosylase |
| A_23_P18384 | 8.05E-04 | 3.3666954 | down | ARMC8 | NM_213654 | armadillo repeat containing 8 |
| A_23_P409888 | 5.23E-04 | 3.3965867 | down | FAM83C | NM_178468 | family with sequence similarity 83. member C |
| A_33_P3269631 | 3.67E-04 | 3.3977277 | down | NAALADL2 | BQ890675 | N-acetylated alpha-linked acidic dipeptidase-like 2 |
| A_23_P393034 | 0.00106518 | 3.4037473 | down | HAS3 | NM_005329 | hyaluronan synthase 3 |
| A_33_P3413335 | 8.40E-05 | 3.4223957 | down |  | AF390550 |  |
| A_23_P334892 | 3.35E-05 | 3.4277637 | down | TMEM102 | NM_178518 | transmembrane protein 102 |
| A_23_P207850 | 4.62E-07 | 3.4325833 | down | TNS4 | NM_032865 | tensin 4 |
| A_24_P19828 | 1.12E-04 | 3.4429195 | down | TOE1 | NM_025077 | target of EGR1. member 1 (nuclear) |
| A_32_P840463 | 8.11E-06 | 3.4922423 | down |  | CR613654 |  |
| A_33_P3230399 | 1.38E-04 | 3.5013847 | down | ZNF784 | NM_203374 | zinc finger protein 784 |
| A_23_P213959 | 7.06E-04 | 3.5212648 | down | PPARGC1B | NM_133263 | peroxisome proliferator-activated receptor gamma. coactivator 1 beta |
| A_23_P345830 | 2.22E-06 | 3.5323672 | down | FASTKD2 | NM_014929 | FAST kinase domains 2 |
| A_23_P302654 | 5.41E-05 | 3.5788603 | down | CEP72 | NM_018140 | centrosomal protein 72kDa |
| A_19_P00320011 | 8.65E-06 | 3.5825408 | down |  |  |  |
| A_33_P3261182 | 3.38E-05 | 3.6624973 | down | C5orf37 | NM_001099271 | chromosome 5 open reading frame 37 |
| A_23_P167194 | 9.31E-06 | 3.7265558 | down | CENPC1 | NM_001812 | centromere protein C 1 |
| A_33_P3418170 | 2.09E-04 | 3.788768 | down | DDX58 | NM_014314 | DEAD (Asp-Glu-Ala-Asp) box polypeptide 58 |
| A_23_P110531 | 2.30E-04 | 3.7975316 | down | FST | NM_013409 | follistatin |
| A_33_P3233774 | 1.86E-05 | 3.851674 | down | ZNF174 | NM_001032292 | zinc finger protein 174 |
| A_23_P259127 | 1.52E-07 | 3.8976061 | down | ESRP1 | NM_017697 | epithelial splicing regulatory protein 1 |
| A_24_P321634 | 3.88E-06 | 3.960867 | down | ZMYM5 | NM_001039650 | zinc finger. MYM-type 5 |
| A_23_P139786 | 1.97E-06 | 3.965415 | down | OASL | NM_003733 | 2'-5'-oligoadenylate synthetase-like |
| A_23_P142239 | 1.57E-04 | 4.0100937 | down | YIF1B | NM_033557 | Yip1 interacting factor homolog B (S. cerevisiae) |
| A_23_P65208 | 4.22E-05 | 4.1224933 | down | ZMYM5 | NM_001039650 | zinc finger. MYM-type 5 |
| A_33_P3306113 | 2.76E-05 | 4.1247263 | down | JMJD4 | NM_023007 | jumonji domain containing 4 |
| A_33_P3263232 | 1.47E-04 | 4.203309 | down | LRRC3 | NM_030891 | leucine rich repeat containing 3 |
| A_23_P402733 | 3.79E-05 | 4.206682 | down | SON | NM_032195 | SON DNA binding protein |
| A_23_P52266 | 2.24E-04 | 4.238457 | down | IFIT1 | NM_001548 | interferon-induced protein with tetratricopeptide repeats 1 |
| A_23_P5568 | 2.24E-05 | 4.332945 | down | SFT2D3 | NM_032740 | SFT2 domain containing 3 |
| A_23_P152038 | 1.05E-04 | 4.370458 | down | TUBGCP5 | NM_052903 | tubulin. gamma complex associated protein 5 |
| A_33_P3816688 | 1.72E-04 | 4.4273586 | down | PPARGC1B | BX648997 | peroxisome proliferator-activated receptor gamma. coactivator 1 beta |
| A_33_P3282489 | 1.04E-04 | 4.433871 | down | GCNT1 | NM_001097634 | glucosaminyl (N-acetyl) transferase 1. core 2 (beta-1.6-N-acetylglucosaminyltransferase) |
| A_33_P3250438 | 1.85E-04 | 4.5091496 | down |  |  |  |
| A_32_P192594 | 9.02E-05 | 4.52464 | down | LOC400099 | XR_040794 | hypothetical LOC400099 |
| A_23_P417148 | 9.90E-06 | 5.3662868 | down | RAI1 | NM_030665 | retinoic acid induced 1 |
| A_33_P3364989 | 4.14E-06 | 5.739648 | down | C20orf134 | NM_001024675 | chromosome 20 open reading frame 134 |
| A_24_P170753 | 1.54E-05 | 5.822692 | down |  | BC066974 |  |
| A_33_P3221119 | 1.62E-06 | 6.3725834 | down | GON4L | NM_032292 | gon-4-like (C. elegans) |
| A_23_P377664 | 9.63E-06 | 6.939772 | down | ALS2 | NM_001135745 | amyotrophic lateral sclerosis 2 (juvenile) |

# Table S3. List of the 166 diffentially expressed probes obtained from *t*-test with BH correction between EXPO and HSC (up : EXPO > HSC).

| **List** | **ProbeName 1/8** | **Genbank Accession** | **Description** | **Gene Symbol** | **Fold change absolute** | ***corrected P-*value** | **regulation** |
| --- | --- | --- | --- | --- | --- | --- | --- |
| **List 1 FC >2** | A_33_P3391796 | NM_005450 | noggin | NOG | 4.43 | 0.0282 | up |
|  | A_23_P70448 | NM_005325 | histone cluster 1, H1a | HIST1H1A | 2.92 | 0.0282 | up |
|  | A_24_P241815 | NM_002229 | jun B proto-oncogene | JUNB | 2.74 | 0.0282 | up |
|  | A_23_P215956 | NM_002467 | v-myc myelocytomatosis viral oncogene homolog (avian) | MYC | 2.74 | 0.0282 | up |
|  | A_23_P91390 | NM_000361 | thrombomodulin | THBD | 2.7 | 0.0282 | up |
|  | A_23_P69738 | NM_023940 | RAS-like, family 11, member B | RASL11B | 2.66 | 0.0282 | up |
|  | A_23_P114057 | NM_017789 | sema domain, immunoglobulin domain (Ig), transmembrane domain (TM) and short cytoplasmic domain, (semaphorin) 4C | SEMA4C | 2.58 | 0.0282 | up |
|  | A_24_P305570 | NM_018993 | Ras and Rab interactor 2 | RIN2 | 2.41 | 0.0282 | up |
|  | A_23_P132121 | NM_173354 | salt-inducible kinase 1 | SIK1 | 2.39 | 0.0282 | up |
|  | A_24_P497186 | NM_182972 | interferon regulatory factor 2 binding protein 2 | IRF2BP2 | 2.35 | 0.0282 | up |
|  | A_19_P00324238 |  | lincRNA:chr3:129602510-129611708 reverse strand |  | 2.32 | 0.0282 | up |
|  | A_33_P3280157 | NR_001290 | small nucleolar RNA, C/D box 116-19 | SNORD116-19 | 2.3 | 0.0282 | up |
|  | A_23_P86917 | NM_003824 | Fas (TNFRSF6)-associated via death domain | FADD | 2.25 | 0.0282 | up |
|  | A_23_P404494 | NM_002185 | interleukin 7 receptor | IL7R | 2.2 | 0.0323 | up |
|  | A_24_P241792 | NM_198893 | zinc finger protein 160 | ZNF160 | 2.17 | 0.036 | up |
|  | A_23_P155939 | NM_182524 | zinc finger protein 595 | ZNF595 | 2.17 | 0.0294 | up |
|  | A_23_P106389 | NM_003612 | semaphorin 7A, GPI membrane anchor (John Milton Hagen blood group) | SEMA7A | 2.16 | 0.0294 | up |
|  | A_33_P3339103 | NM_203290 | polymerase (RNA) I polypeptide C, 30kDa | POLR1C | 2.15 | 0.0294 | up |
|  | A_32_P58425 | NM_153812 | PHD finger protein 13 | PHF13 | 2.12 | 0.0294 | up |
|  | A_23_P345674 | NM_021216 | zinc finger protein 71 | ZNF71 | 2.12 | 0.0282 | up |
|  | A_23_P110052 | NM_023067 | forkhead box L2 | FOXL2 | 2.11 | 0.0294 | up |
|  | A_23_P250385 | NM_005322 | histone cluster 1, H1b | HIST1H1B | 2.11 | 0.0347 | up |
|  | A_24_P69095 | NM_003633 | ectodermal-neural cortex (with BTB-like domain) | ENC1 | 2.1 | 0.0282 | up |
|  | A_33_P3362521 | NM_016474 | chromosome 3 open reading frame 19 | C3orf19 | 2.09 | 0.0282 | up |
|  | A_33_P3286953 | NM_197941 | ADAM metallopeptidase with thrombospondin type 1 motif, 6 | ADAMTS6 | 2.07 | 0.0282 | up |
| **List 1 FC >2** | **ProbeName 2/8** | **Genbank Accession** | **Description** | **Gene Symbol** | **Fold change absolute** | ***corrected P-*value** | **regulation** |
|  | A_23_P163117 | NM_024644 | chromosome 14 open reading frame 169 | C14orf169 | 2.04 | 0.0282 | up |
|  | A_23_P169039 | NM_003068 | snail homolog 2 (Drosophila) | SNAI2 | 2.04 | 0.0294 | up |
|  |  |  |  |  |  |  |  |
|  | A_23_P4294 | NM_014519 | zinc finger protein 232 | ZNF232 | 2.04 | 0.0315 | up |
|  | A_19_P00322080 |  | lincRNA:chr19:28284802-28288920 forward strand |  | 2.04 | 0.0347 | up |
|  | A_23_P12896 | NM_022725 | Fanconi anemia, complementation group F | FANCF | 2.03 | 0.0323 | up |
|  | A_23_P99540 | NM_004926 | zinc finger protein 36, C3H type-like 1 | ZFP36L1 | 2.03 | 0.0282 | up |
|  | A_32_P20523 | NM_145715 | tigger transposable element derived 2 | TIGD2 | 2.02 | 0.0294 | up |
|  | A_32_P43465 | NM_006962 | zinc finger protein 182 | ZNF182 | 2.02 | 0.0059 | up |
|  | A_24_P68019 | NM_138347 | zinc finger protein 551 | ZNF551 | 2.02 | 0.036 | up |
|  | A_33_P3220723 | NM_015196 | KIAA0922 | KIAA0922 | 2.01 | 0.0294 | up |
|  | A_33_P3393796 | NM_020810 | TRM5 tRNA methyltransferase 5 homolog (S. cerevisiae) | TRMT5 | 2.01 | 0.0347 | up |

|  | **ProbeName 3/8** | **Genbank Accession** | **Description** | **Gene Symbol** | **Fold change absolute** | ***corrected P-*value** | **regulation** |
| --- | --- | --- | --- | --- | --- | --- | --- |
| **List 2 2>FC >1.5** | A_24_P911676 | NM_003107 | SRY (sex determining region Y)-box 4 | SOX4 | 2 | 0.0344 | up |
|  | A_23_P101960 | NM_006887 | zinc finger protein 36, C3H type-like 2 | ZFP36L2 | 2 | 0.0282 | up |
|  | A_33_P3246083 | NM_017897 | 3-oxoacyl-ACP synthase, mitochondrial | OXSM | 1.99 | 0.0347 | up |
|  | A_19_P00808471 |  | lincRNA:chr5:121370601-121377751 forward strand |  | 1.99 | 0.0372 | up |
|  | A_33_P3294881 | NM_016474 | chromosome 3 open reading frame 19 | C3orf19 | 1.97 | 0.0324 | up |
|  | A_23_P166536 | NM_014577 | bromodomain containing 1 | BRD1 | 1.96 | 0.0305 | up |
|  | A_23_P132263 | NM_019843 | eukaryotic translation initiation factor 4E nuclear import factor 1 | EIF4ENIF1 | 1.96 | 0.0365 | up |
|  | A_23_P153197 | NM_170695 | TGFB-induced factor homeobox 1 | TGIF1 | 1.96 | 0.0282 | up |
|  | A_23_P206077 | NM_022767 | apoptosis enhancing nuclease | AEN | 1.95 | 0.0294 | up |
|  | A_23_P63153 | NM_007204 | DEAD (Asp-Glu-Ala-Asp) box polypeptide 20 | DDX20 | 1.95 | 0.0294 | up |
|  | A_23_P210763 | NM_000214 | jagged 1 (Alagille syndrome) | JAG1 | 1.95 | 0.0282 | up |
|  | A_32_P112623 | XR_079078 | hypothetical LOC100293193 | LOC100293193 | 1.94 | 0.0185 | up |
|  | A_33_P3237729 | NM_003687 | PDZ and LIM domain 4 | PDLIM4 | 1.94 | 0.036 | up |
|  | A_23_P20722 | NM_003086 | small nuclear RNA activating complex, polypeptide 4, 190kDa | SNAPC4 | 1.94 | 0.0401 | up |
|  | A_23_P146187 | NM_015169 | RRS1 ribosome biogenesis regulator homolog (S. cerevisiae) | RRS1 | 1.93 | 0.0282 | up |
|  | A_23_P164559 | NM_032436 | zinc finger protein 828 | ZNF828 | 1.93 | 0.0408 | up |
|  | A_33_P3309924 | NM_001527 | histone deacetylase 2 | HDAC2 | 1.92 | 0.036 | up |
|  | A_33_P3423859 | NM_006242 | protein phosphatase 1, regulatory (inhibitor) subunit 3D | PPP1R3D | 1.92 | 0.0282 | up |
|  | A_23_P30307 | NM_004270 | mediator complex subunit 7 | MED7 | 1.91 | 0.0401 | up |
|  | A_23_P200801 | NM_001002811 | phosphodiesterase 4D interacting protein | PDE4DIP | 1.91 | 0.0282 | up |
|  | A_33_P3877728 | BX647090 | Smith-Magenis syndrome chromosome region, candidate 6 | SMCR6 | 1.91 | 0.0323 | up |
|  | A_23_P69670 | NM_018366 | cappuccino homolog (mouse) | CNO | 1.9 | 0.0294 | up |
|  | A_23_P347508 | NM_023077 | chromosome 1 open reading frame 163 | C1orf163 | 1.89 | 0.0324 | up |
|  | A_24_P835500 | NM_001034841 | inositol 1,4,5-triphosphate receptor interacting protein-like 2 | ITPRIPL2 | 1.89 | 0.0367 | up |
|  | A_23_P41066 | NM_170713 | Ras association (RalGDS/AF-6) domain family member 1 | RASSF1 | 1.89 | 0.0282 | up |
|  | A_19_P00803393 |  | lincRNA:chr5:44826637-44828692 forward strand |  | 1.89 | 0.0401 | up |
|  | A_33_P3251685 | NM_020786 | pyruvate dehyrogenase phosphatase catalytic subunit 2 | PDP2 | 1.88 | 0.036 | up |
|  | **ProbeName 4/8** | **Genbank Accession** | **Description** | **Gene Symbol** | **Fold change absolute** | ***corrected P-*value** | **regulation** |
| **List 2 2>FC >1.5** | A_23_P111303 | NM_014892 | RNA binding motif protein 16 | RBM16 | 1.88 | 0.0282 | up |
|  | A_23_P159101 | NM_022717 | small nuclear ribonucleoprotein 35kDa (U11/U12) | SNRNP35 | 1.88 | 0.0294 | up |
|  | A_24_P96961 | NM_025106 | splA/ryanodine receptor domain and SOCS box containing 1 | SPSB1 | 1.88 | 0.0347 | up |
|  | A_33_P3253264 | NM_005341 | zinc finger and BTB domain containing 48 | ZBTB48 | 1.88 | 0.0323 | up |
|  | A_33_P3290487 | NM_024706 | zinc finger protein 668 | ZNF668 | 1.88 | 0.0294 | up |
|  | A_33_P3279831 | NM_006813 | proline-rich nuclear receptor coactivator 1 | PNRC1 | 1.87 | 0.0407 | up |
|  | A_33_P3257678 | NM_001005464 | histone cluster 2, H3a | HIST2H3A | 1.86 | 0.0424 | up |
|  | A_33_P3358977 | NM_032193 | ribonuclease H2, subunit C | RNASEH2C | 1.86 | 0.036 | up |
|  | A_23_P130466 | NM_021089 | zinc finger protein 8 | ZNF8 | 1.86 | 0.0324 | up |
|  | A_24_P74064 | NM_003409 | zinc finger protein 161 homolog (mouse) | ZFP161 | 1.85 | 0.036 | up |
|  | A_33_P3394183 | NM_207395 | zinc finger protein 324B | ZNF324B | 1.85 | 0.0294 | up |
|  | A_23_P413634 | NM_024620 | zinc finger protein 329 | ZNF329 | 1.85 | 0.0372 | up |
|  | A_23_P213319 | NM_197941 | ADAM metallopeptidase with thrombospondin type 1 motif, 6 | ADAMTS6 | 1.84 | 0.0424 | up |
|  | A_33_P3395971 | NM_145232 | cytosolic thiouridylase subunit 1 homolog (S. pombe) | CTU1 | 1.84 | 0.0382 | up |
|  | A_23_P301247 | NM_003517 | histone cluster 2, H2ac | HIST2H2AC | 1.84 | 0.0347 | up |
|  | A_19_P00316946 |  | lincRNA:chr19:28269547-28283939 reverse strand |  | 1.84 | 0.0365 | up |
|  | A_19_P00320427 |  | lincRNA:chr19:28284466-28288902 forward strand |  | 1.84 | 0.0407 | up |
|  | A_24_P506977 | NR_003697 | chromosome 7 open reading frame 40 | C7orf40 | 1.83 | 0.0282 | up |
|  | A_24_P854913 | NM_001127395 | family with sequence similarity 119, member A | FAM119A | 1.83 | 0.0499 | up |
|  | A_33_P3233645 | NM_005950 | metallothionein 1G | MT1G | 1.83 | 0.0464 | up |
|  | A_23_P255523 | NM_017621 | alkB, alkylation repair homolog 4 (E. coli) | ALKBH4 | 1.82 | 0.0347 | up |
|  | A_33_P3355503 | NM_005250 | forkhead box L1 | FOXL1 | 1.82 | 0.036 | up |
|  | A_33_P3376140 | NM_170736 | potassium inwardly-rectifying channel, subfamily J, member 15 | KCNJ15 | 1.82 | 0.0282 | up |
|  | A_33_P3262670 | AL832120 | MAP7 domain-containing protein 3 [Source:UniProtKB/Swiss-Prot;Acc:Q8IWC1] [ENST00000370661] | MAP7 domain-containing p 3 | 1.82 | 0.0347 | up |
|  | A_23_P152235 | NM_024336 | iroquois homeobox 3 | IRX3 | 1.81 | 0.0315 | up |
|  | **ProbeName 5/8** | **Genbank Accession** | **Description** | **Gene Symbol** | **Fold change absolute** | ***corrected P-*value** | **regulation** |
| List 2 2>FC >1.5 | A_23_P214222 | NM_002356 | myristoylated alanine-rich protein kinase C substrate | MARCKS | 1.81 | 0.0367 | up |
|  | A_33_P3338335 | NM_177401 | midnolin | MIDN | 1.81 | 0.0282 | up |
|  | A_33_P3398091 | NM_001130031 | zinc finger protein 562 | ZNF562 | 1.81 | 0.0367 | up |
|  | A_19_P00320136 |  | lincRNA:chr15:25332807-25334252 forward strand |  | 1.81 | 0.0323 | up |
|  | A_24_P108311 | NM_015277 | neural precursor cell expressed, developmentally down-regulated 4-like | NEDD4L | 1.8 | 0.0323 | up |
|  | A_33_P3377519 | NM_024014 | homeobox A6 | HOXA6 | 1.79 | 0.0324 | up |
|  | A_33_P3666797 | AL832853 | hypothetical protein LOC144874 | LOC144874 | 1.79 | 0.0401 | up |
|  | A_24_P413126 | NM_020182 | prostate transmembrane protein, androgen induced 1 | PMEPA1 | 1.79 | 0.0347 | up |
|  | A_23_P12272 | NM_138558 | protein phosphatase 1, regulatory (inhibitor) subunit 8 | PPP1R8 | 1.79 | 0.0323 | up |
|  | A_23_P26847 | NM_000346 | SRY (sex determining region Y)-box 9 | SOX9 | 1.79 | 0.0282 | up |
|  | A_23_P168828 | NM_005655 | Kruppel-like factor 10 | KLF10 | 1.78 | 0.0294 | up |
|  | A_24_P782308 | NM_001144967 | neural precursor cell expressed, developmentally down-regulated 4-like | NEDD4L | 1.78 | 0.0499 | up |
|  | A_23_P150080 | NM_183005 | ribonuclease P/MRP 38kDa subunit | RPP38 | 1.78 | 0.0282 | up |
|  | A_33_P3224307 | NR_003010 | small Cajal body-specific RNA 12 | SCARNA12 | 1.78 | 0.0456 | up |
|  | A_23_P383986 | NM_015892 | carbohydrate (N-acetylgalactosamine 4-sulfate 6-O) sulfotransferase 15 | CHST15 | 1.77 | 0.0452 | up |
|  | A_23_P70409 | NM_004875 | polymerase (RNA) I polypeptide C, 30kDa | POLR1C | 1.77 | 0.0367 | up |
|  | A_19_P00324814 |  | lincRNA:chrX:73164675-73232924 forward strand |  | 1.77 | 0.0415 | up |
|  | A_33_P3346866 | NM_017822 | chromosome 12 open reading frame 41 | C12orf41 | 1.76 | 0.0323 | up |
|  | A_23_P76901 | NM_015549 | pleckstrin homology domain containing, family G (with RhoGef domain) member 3 | PLEKHG3 | 1.76 | 0.0401 | up |
|  | A_23_P26314 | NM_020807 | zinc finger protein 319 | ZNF319 | 1.76 | 0.0372 | up |
|  | A_33_P3262495 | NM_032772 | zinc finger protein 503 | ZNF503 | 1.76 | 0.032 | up |
|  | A_33_P3386364 | NM_022725 | Fanconi anemia, complementation group F | FANCF | 1.75 | 0.0294 | up |
|  | A_24_P202567 | NM_025194 | inositol 1,4,5-trisphosphate 3-kinase C | ITPKC | 1.75 | 0.0441 | up |
|  | A_23_P202737 | NM_003772 | jerky homolog-like (mouse) | JRKL | 1.75 | 0.0305 | up |

|  | **ProbeName 6/8** | **Genbank Accession** | **Description** | **Gene Symbol** | **Fold change absolute** | ***corrected P-*value** | **regulation** |
| --- | --- | --- | --- | --- | --- | --- | --- |
| List 2 2>FC >1.5 | A_23_P103104 | NM_002405 | MFNG O-fucosylpeptide 3-beta-N-acetylglucosaminyltransferase | MFNG | 1.75 | 0.0347 | up |
|  | A_23_P141960 | NM_203344 | SERTA domain containing 3 | SERTAD3 | 1.75 | 0.0347 | up |
|  | A_23_P202458 | NM_006963 | zinc finger protein 22 (KOX 15) | ZNF22 | 1.75 | 0.036 | up |
|  | A_33_P3369761 | NM_001161779 | pyruvate dehyrogenase phosphatase catalytic subunit 1 | PDP1 | 1.74 | 0.036 | up |
|  | A_33_P3380982 | NM_018247 | transmembrane protein 30A | TMEM30A | 1.74 | 0.0424 | up |
|  | A_23_P3856 | NM_153688 | zinc finger protein 1 homolog (mouse) | ZFP1 | 1.74 | 0.0409 | up |
|  | A_33_P3230399 | NM_203374 | zinc finger protein 784 | ZNF784 | 1.74 | 0.0441 | up |
|  | A_23_P68505 |  | chromosome 20 open reading frame 177 | C20orf177 | 1.73 | 0.0372 | up |
|  | A_23_P119964 | NM_005760 | CCAAT/enhancer binding protein (C/EBP), zeta | CEBPZ | 1.73 | 0.0408 | up |
|  | A_23_P78152 | NM_024039 | MIS12, MIND kinetochore complex component, homolog (S. pombe) | MIS12 | 1.73 | 0.0372 | up |
|  | A_23_P386411 | NM_005038 | peptidylprolyl isomerase D | PPID | 1.73 | 0.0441 | up |
|  | A_23_P340890 | CR601567 | THAP domain-containing protein 3 [Source:UniProtKB/Swiss-Prot;Acc:Q8WTV1] [ENST00000307896] | THAP domain-containing P 3 | 1.73 | 0.0456 | up |
|  | A_32_P131050 | NM_021964 | zinc finger protein 148 | ZNF148 | 1.73 | 0.0424 | up |
|  | A_33_P3368139 | NM_005921 | mitogen-activated protein kinase kinase kinase 1 | MAP3K1 | 1.72 | 0.0379 | up |
|  | A_23_P37654 | NM_001164273 | MAX gene associated | MGA | 1.72 | 0.0323 | up |
|  | A_33_P3394234 | NM_003434 | zinc finger protein 133 | ZNF133 | 1.72 | 0.0408 | up |
|  | A_33_P3240512 | NM_138444 | potassium channel tetramerisation domain containing 12 | KCTD12 | 1.71 | 0.0441 | up |
|  | A_33_P3358208 | NM_013358 | peptidyl arginine deiminase, type I | PADI1 | 1.71 | 0.0408 | up |
|  | A_33_P3304212 | NM_015549 | pleckstrin homology domain containing, family G (with RhoGef domain) member 3 | PLEKHG3 | 1.71 | 0.0401 | up |
|  | A_23_P331813 | NM_020832 | zinc finger protein 687 | ZNF687 | 1.71 | 0.0347 | up |
|  | A_32_P19716 | NM_001080470 | zinc finger protein 697 | ZNF697 | 1.71 | 0.0392 | up |
|  | A_33_P3315410 | NM_004645 | coilin | COIL | 1.7 | 0.0495 | up |
|  | A_33_P3409477 | NM_032873 | ubiquitin associated and SH3 domain containing, B | UBASH3B | 1.7 | 0.0395 | up |
|  | A_32_P67623 | NM_017848 | family with sequence similarity 120C | FAM120C | 1.69 | 0.0401 | up |

|  | **ProbeName 7/8** | **Genbank Accession** | **Description** | **Gene Symbol** | **Fold change absolute** | ***corrected P-*value** | **regulation** |
| --- | --- | --- | --- | --- | --- | --- | --- |
| List 2  2>FC >1.5 | A_23_P47885 | NM_153377 | leucine-rich repeats and immunoglobulin-like domains 3 | LRIG3 | 1.69 | 0.0401 | up |
|  | A_23_P15603 | NM_024864 | mitochondrial rRNA methyltransferase 1 homolog (S. cerevisiae) | MRM1 | 1.68 | 0.0383 | up |
|  | A_19_P00804590 |  | lincRNA:chr9:2739400-2746875 reverse strand |  | 1.68 | 0.0424 | up |
|  | A_33_P3219785 | NM_023016 | ankyrin repeat domain 57 | ANKRD57 | 1.67 | 0.0407 | up |
|  | A_23_P7976 | NM_005321 | histone cluster 1, H1e | HIST1H1E | 1.67 | 0.0415 | up |
|  | A_23_P212617 | NM_003234 | transferrin receptor (p90, CD71) | TFRC | 1.67 | 0.0398 | up |
|  | A_33_P3285047 | NM_153028 | zinc finger protein 75a | ZNF75A | 1.67 | 0.0401 | up |
|  | A_24_P133488 | NM_017955 | cell division cycle associated 4 | CDCA4 | 1.66 | 0.0401 | up |
|  | A_32_P135336 | NR_002556 | coiled-coil domain containing 101 pseudogene | LOC388242 | 1.66 | 0.0404 | up |
|  | A_23_P64669 | NM_017612 | zinc finger, CCHC domain containing 8 | ZCCHC8 | 1.66 | 0.0401 | up |
|  | A_19_P00800033 |  | lincRNA:chr8:106166599-106275374 forward strand |  | 1.66 | 0.0483 | up |
|  | A_23_P364544 | NM_175874 | chromosome 12 open reading frame 60 | C12orf60 | 1.65 | 0.0499 | up |
|  | A_32_P221799 | NM_003514 | histone cluster 1, H2am | HIST1H2AM | 1.65 | 0.0424 | up |
|  | A_23_P395374 | NM_003539 | histone cluster 1, H4d | HIST1H4D | 1.64 | 0.0464 | up |
|  | A_23_P82913 | NM_017956 | tRNA methyltransferase 12 homolog (S. cerevisiae) | TRMT12 | 1.64 | 0.0441 | up |
|  | A_23_P32253 | NM_005384 | nuclear factor, interleukin 3 regulated | NFIL3 | 1.63 | 0.0464 | up |

| list | **ProbeName 8/8** | **Genbank Accession** | **Description** | **Gene Symbol** | **Fold change absolute** | ***corrected P-*value** | **regulation** |
| --- | --- | --- | --- | --- | --- | --- | --- |
| List 2 2>FC >1.5 | A_23_P65618 | NM_000359 | transglutaminase 1 (K polypeptide epidermal type I, protein-glutamine-gamma-glutamyltransferase) | TGM1 | 1.96 | 0.0315 | down |
|  | A_33_P3211513 | NM_001162407 | CDC-like kinase 1 | CLK1 | 1.92 | 0.0377 | down |
|  | A_19_P00322824 |  | lincRNA:chr7:32953392-32954696 reverse strand |  | 1.9 | 0.0441 | down |
|  | A_23_P384532 | NM_145020 | coiled-coil domain containing 11 | CCDC11 | 1.85 | 0.0372 | down |
|  | A_32_P198303 | NM_181706 | DnaJ (Hsp40) homolog, subfamily C, member 24 | DNAJC24 | 1.85 | 0.0324 | down |
|  | A_23_P137470 | NM_020808 | signal-induced proliferation-associated 1 like 2 | SIPA1L2 | 1.77 | 0.0324 | down |
|  | A_33_P3413997 |  |  |  | 1.76 | 0.0323 | down |
|  | A_33_P3346688 |  |  |  | 1.74 | 0.0464 | down |
|  | A_33_P3212570 |  |  |  | 1.73 | 0.0492 | down |
|  | A_33_P3236392 | NM_030916 | poliovirus receptor-related 4 | PVRL4 | 1.72 | 0.0441 | down |
|  | A_24_P55496 | NM_053001 | odd-skipped related 2 (Drosophila) | OSR2 | 1.69 | 0.04 | down |
|  | A_23_P164912 | NM_022165 | lin-7 homolog B (C. elegans) | LIN7B | 1.68 | 0.0372 | down |
|  | A_23_P79302 | NM_177964 | LY6/PLAUR domain containing 6B | LYPD6B | 1.68 | 0.0461 | down |
